# Supplementary material for: Point-of-care early infant HIV diagnosis at birth in a pragmatic cluster-randomized trial in Mozambique and Tanzania: A comparative cost and cost-effectiveness study
Source: PLoS Med. 2026 May 6;23(5):e1005069. doi: 10.1371/journal.pmed.1005069 (PMC13166934; doi:10.1371/journal.pmed.1005069)
Supplement: S1 Text — Fig A: Violin plots of per site time from birth to PoC HIV EID testing and treatment initiation. (A) First EID test among all HIV-exposed infants; (B) ART initiation among infants diagnosed with HIV up to 16 weeks of age. Only sites with infants with HIV are shown. Black triangle markers represent individual data. VEID, very early infant (HIV) diagnosis; SoC = standard of care; EID, early infant (HIV) diagnosis; ART, antiretroviral treatment. Created in BioRender. Hoelscher, M. (2025) https://BioRender.com/u260ruo. Fig B: Per site point-of-care early infant (HIV) diagnosis test cost components. (A) Cost estimates expressed in 2020 US$. (B) Proportion of total cost per test. Fig C: Per site deterministic one-way sensitivity analysis showing influence of key assumptions on cost per test. Per test cost difference is shown on the x-axis and parameters with ranges evaluated are shown on the y-axis. High and low colors indicate the direction in which the parameter varies. Table A: Point-of-care early infant (HIV) diagnosis test cost components. Cost estimates are expressed in 2020 US$. Sites are categorized as high (>12 tests per week), medium (5–12 tests per week), and low (<5 tests per week) testing volume. Equipment includes amortized costs of initial purchase, installation, and yearly maintenance of PoC analyzers. Overhead includes apportioned costs of electricity, communications, and facility upgrades. Table B: Modeled incremental cost and effect estimates for 1-week ART uptake per infant diagnosed with HIV. Estimated means and 95% CrI shown. SoC = standard of care; VEID, very early infant (HIV) diagnosis; CrI = credible interval. Table C: Modeled incremental cost and effect estimates for 8-week PoC EID uptake per infant exposed to HIV. Estimated means and 95% CrI shown. SoC = standard of care; VEID, very early infant (HIV) diagnosis; CrI = credible interval. Fig D: Histogram of median PoC tests per ISO week with divisions for low-, medium-, and high-volume sites sho [file pmed.1005069.s001.pdf]

Supplementary material

Point-of-care early infant diagnosis at birth in a pragmatic cluster-randomized trial in Mozambique and Tanzania: a comparative cost and cost-effectiveness study

Kira Elsbernd[1,2,3], Issa Sabi[4], Ilesh Jani[5], Chishamiso Mudenyanga[6], Siriel Boniface[4], Arlete Mahumane[5], Joaquim Lequechane[5], Falume Chale[5], Bindiya Meggi[5], Kassia Pereira[5], Raphael Edom[4], Anange F. Lwilla[4], W. Chris Buck[7], Nyanda Elias Ntinyinya[4], Michael Hölscher[1,3,8,9], Till Bärnighausen[10,11], Arne Kroidl[1,3], and Stefan Kohler[10,11,12]

[1]*Institute of Infectious Diseases and Tropical Medicine, LMU University Hospital, LMU Munich, Munich, Germany*

[2]*Institute for Medical Information Processing, Biometry, and Epidemiology, LMU Munich, Munich, Germany*

[3]*German Center for Infection Research (DZIF), partner site Munich, Germany*

[4]*National Institute for Medical Research, Mbeya, Tanzania*

[5]*Instituto Nacional de Saúde, Mozambique*

[6]*Clinton Health Access Initiative, Mozambique*

[7]*University of California Los Angeles, David Geffen School of Medicine, USA*

[8]*Fraunhofer Institute for Translational Medicine and Pharmacology ITMP, Immunology, Infection and Pandemic Research, Munich, Germany*

[9]*Unit Global Health, Helmholtz Center Munich, German Research Center for Environmental Health (HMGU), Neuherberg, Germany*

[10]*Heidelberg Institute of Global Health, Faculty of Medicine and University Hospital, Heidelberg University, Heidelberg, Germany*

[11]*German Center for Infection Research (DZIF), partner site Heidelberg, Germany*

[12]*Institute of Social Medicine, Epidemiology and Health Economics, Charité – Universitätsmedizin Berlin, Corporate Member of Freie Universität Berlin and Humboldt-Universität zu Berlin, Berlin, Germany*

Contents

1 Supplementary Figures 2

2 Supplementary Tables 5

3 Supplementary methods: PoC testing volume 6

4 Supplementary methods: costing 6

5 Supplementary methods: economic model 8

1 Supplementary Figures

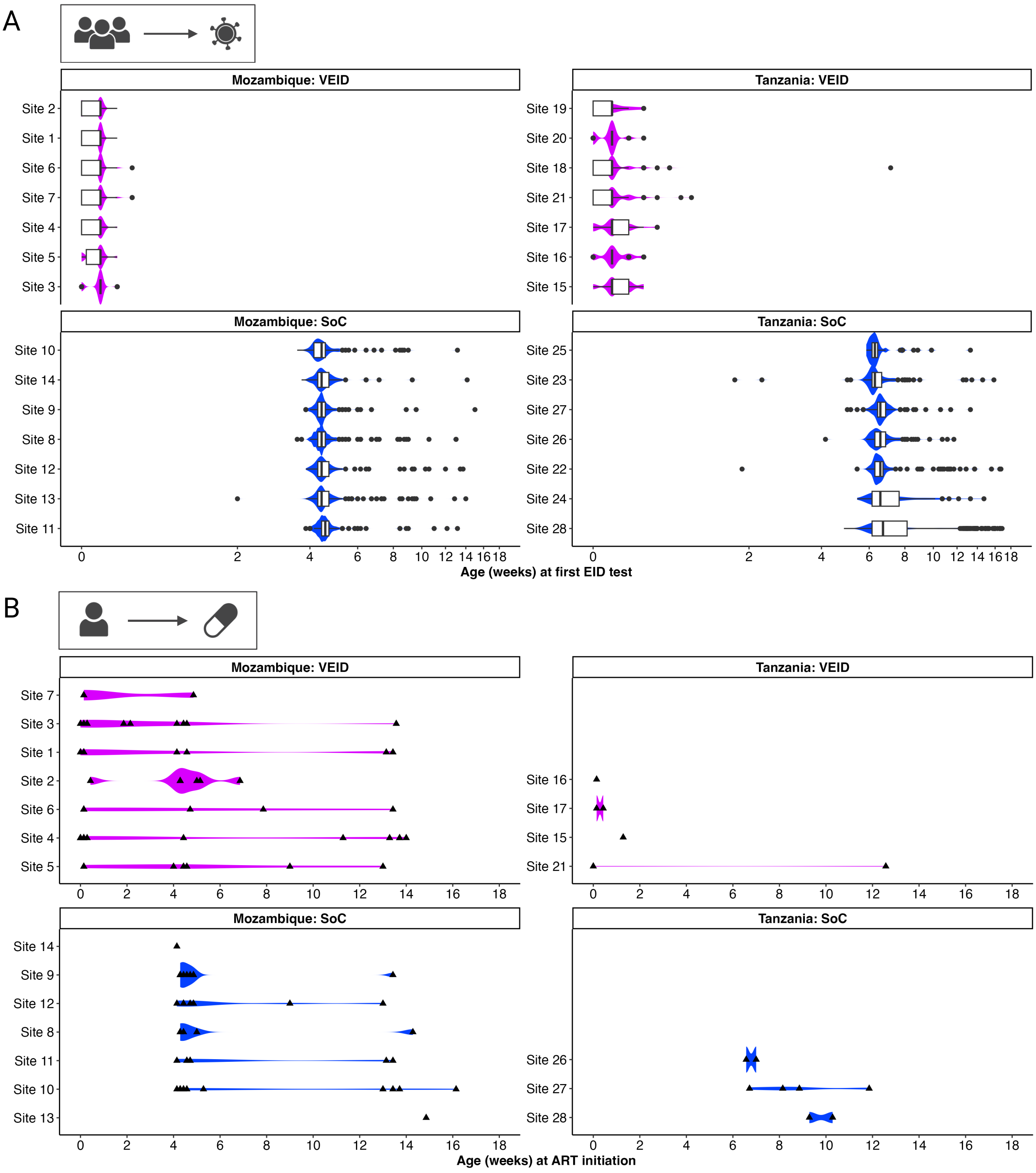

Fig A: Violin plots of per site time from birth to PoC EID testing and treatment initiation. (A) First EID test among all infants exposed to HIV; (B) ART initiation among infants diagnosed with HIV up to 16 weeks of age. Only sites with infants diagnosed with HIV are shown. Black triangle markers represent individual data. *VEID = very early infant (HIV) diagnosis; SoC = standard of care; EID = early infant (HIV) diagnosis; ART = antiretroviral treatment. Created in BioRender. Hoelscher, M. (2025) https://BioRender.com/u260ruo.*

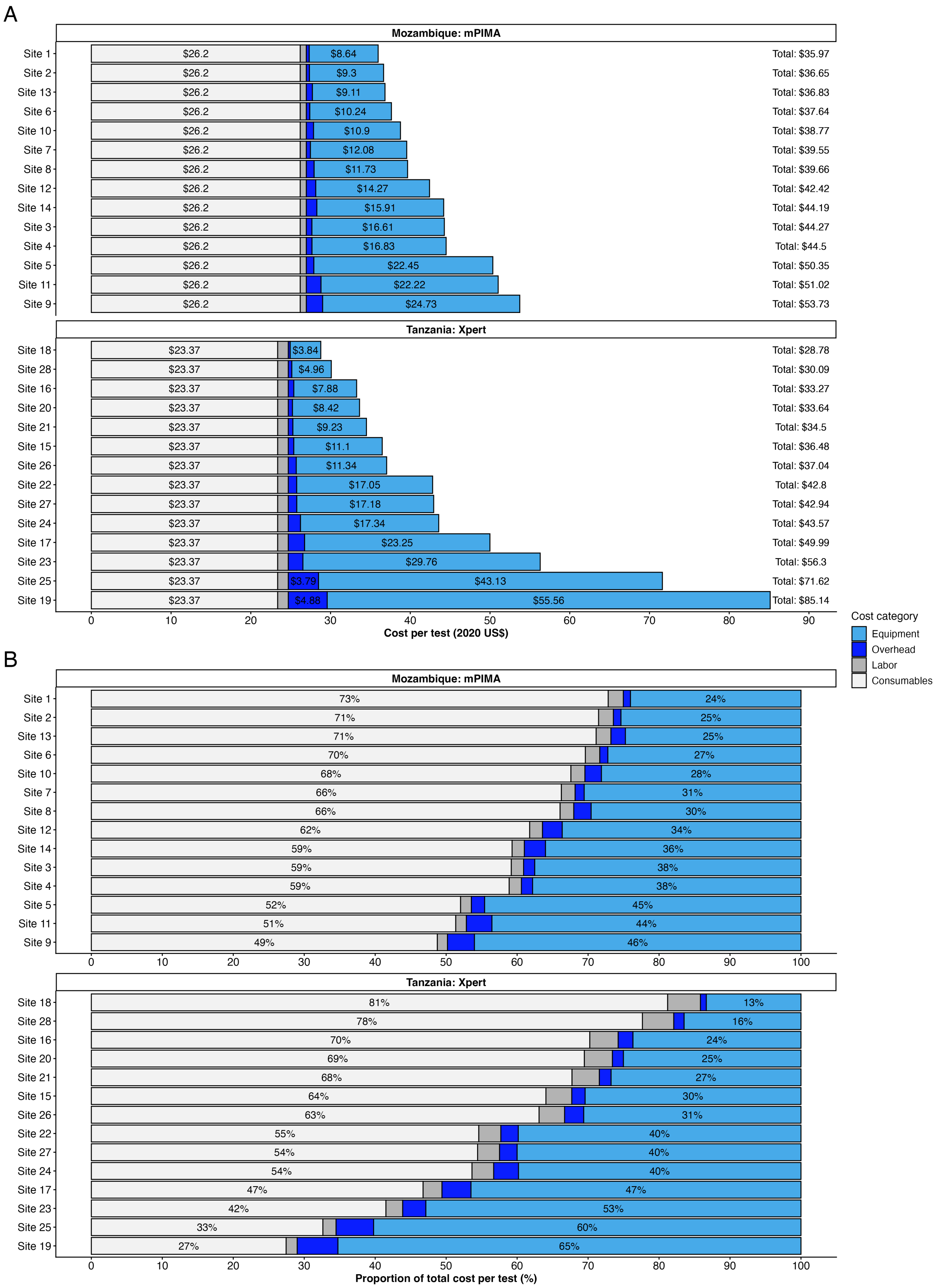

Fig B: Per site point-of-care early infant (HIV) diagnosis test cost components. (A) Cost estimates expressed in 2020 US$. (B) Proportion of total cost per test.

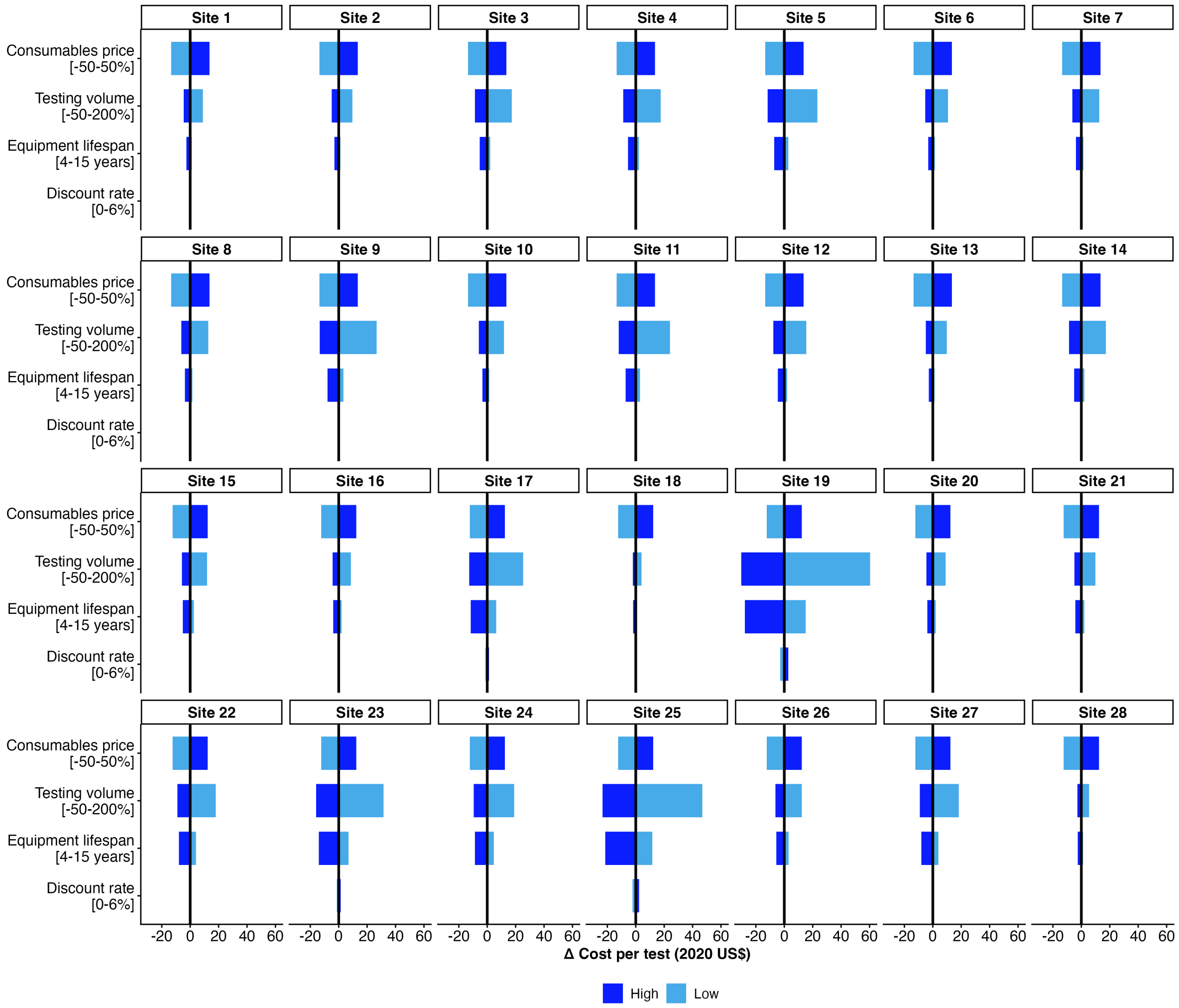

Fig C: Per site deterministic one-way sensitivity analysis showing influence of key assumptions on cost per test. Per test cost difference is shown on the x-axis and parameters with ranges evaluated are shown on the y-axis. High and low colors indicate the direction in which the parameter varies.

2 Supplementary Tables

Site	Country: PoC platform	PoC volume category	Equipment	Overhead	Labor	Consumables
Site 1	Mozambique: mPIMA	High (> 12)	$8.64	$0.36	$0.77	$26.20
Site 2	Mozambique: mPIMA	High (> 12)	$9.30	$0.39	$0.77	$26.20
Site 3	Mozambique: mPIMA	High (> 12)	$16.61	$0.66	$0.77	$26.20
Site 4	Mozambique: mPIMA	Med. (5–12)	$16.83	$0.70	$0.77	$26.20
Site 5	Mozambique: mPIMA	Med. (5–12)	$22.45	$0.94	$0.77	$26.20
Site 6	Mozambique: mPIMA	High (> 12)	$10.24	$0.43	$0.77	$26.20
Site 7	Mozambique: mPIMA	High (> 12)	$12.08	$0.50	$0.77	$26.20
Site 8	Mozambique: mPIMA	Med. (5–12)	$11.73	$0.97	$0.77	$26.20
Site 9	Mozambique: mPIMA	Low (< 5)	$24.73	$2.04	$0.77	$26.20
Site 10	Mozambique: mPIMA	Med. (5–12)	$10.90	$0.90	$0.77	$26.20
Site 11	Mozambique: mPIMA	Low (< 5)	$22.22	$1.83	$0.77	$26.20
Site 12	Mozambique: mPIMA	Med. (5–12)	$14.27	$1.18	$0.77	$26.20
Site 13	Mozambique: mPIMA	Med. (5–12)	$9.11	$0.75	$0.77	$26.20
Site 14	Mozambique: mPIMA	Med. (5–12)	$15.91	$1.31	$0.77	$26.20
Site 15	Tanzania: Xpert	Med. (5–12)	$11.10	$0.68	$1.33	$23.37
Site 16	Tanzania: Xpert	Med. (5–12)	$7.88	$0.69	$1.33	$23.37
Site 17	Tanzania: Xpert	Low (< 5)	$23.25	$2.04	$1.33	$23.37
Site 18	Tanzania: Xpert	High (> 12)	$3.84	$0.24	$1.33	$23.37
Site 19	Tanzania: Xpert	Low (< 5)	$55.56	$4.88	$1.33	$23.37
Site 20	Tanzania: Xpert	Med. (5–12)	$8.42	$0.52	$1.33	$23.37
Site 21	Tanzania: Xpert	Med. (5–12)	$9.23	$0.57	$1.33	$23.37
Site 22	Tanzania: Xpert	Med. (5–12)	$17.05	$1.05	$1.33	$23.37
Site 23	Tanzania: Xpert	Low (< 5)	$29.76	$1.83	$1.33	$23.37
Site 24	Tanzania: Xpert	Low (< 5)	$17.34	$1.52	$1.33	$23.37
Site 25	Tanzania: Xpert	Low (< 5)	$43.13	$3.79	$1.33	$23.37
Site 26	Tanzania: Xpert	Med. (5–12)	$11.34	$1.00	$1.33	$23.37
Site 27	Tanzania: Xpert	Med. (5–12)	$17.18	$1.06	$1.33	$23.37
Site 28	Tanzania: Xpert	High (> 12)	$4.96	$0.44	$1.33	$23.37

Table A: Point-of-care early infant (HIV) diagnosis test cost components. Cost estimates are expressed in 2020 US$. Sites are categorized as high (> 12 tests per week), medium (5–12 tests per week), and low (< 5 tests per week) testing volume. Equipment includes amortized costs of initial purchase, installation, and yearly maintenance of PoC analyzers. Overhead includes apportioned costs of electricity, communications, and facility upgrades.

Country	Study arm	Effect mean (95% CrI)	Cost mean (95% CrI)
Mozambique	SoC	..	$3566 (3122, 4053)
Mozambique	VEID	0.900 (0.675, 0.985)	$6197 (5415, 7059)
Tanzania	SoC	..	$8167 (7153, 9285)
Tanzania	VEID	0.599 (0.214, 0.895)	$14433 (12620, 16417)

Table B: Modeled incremental cost and effect estimates for 1-week ART uptake per infant diagnosed with HIV. Estimated means and 95% CrI shown. *SoC = standard of care; VEID = very early infant (HIV) diagnosis; CrI = credible interval.*

Country	Study arm	Effect mean (95% CrI)	Cost mean (95% CrI)
Mozambique	SoC	0.913 (0.877, 0.941)	$37.23 (32.40, 42.49)
Mozambique	VEID	0.993 (0.989, 0.995)	$87.95 (76.35, 100.71)
Tanzania	SoC	0.785 (0.712, 0.848)	$40.84 (35.55, 46.63)
Tanzania	VEID	0.995 (0.992, 0.996)	$69.36 (60.30, 79.38)

Table C: Modeled incremental cost and effect estimates for 8-week PoC EID uptake per infant exposed to HIV. Estimated means and 95% CrI shown. *SoC = standard of care; VEID = very early infant (HIV) diagnosis; CrI = credible interval.*

3 Supplementary methods: PoC testing volume

3.1 PoC testing capacity

We assumed a 248-day work year and 38-hour work week. Run times for PoC EID assays were 52 minutes for Abbott mPIMA HIV-1/2 Detect [1] and 90 minutes for Cepheid Xpert HIV-1 Qual [2]. Including the time to prepare and start each test reported anonymously by study nurses, PoC testing capacities per health facility were calculated as 32 tests per week for mPIMA, 44 tests per week for Xpert II, and 88 tests per week for Xpert IV. All VEID sites in Mozambique had two mPIMA analyzers for a capacity of 72 tests per week.

3.2 Categorization of PoC testing volume

For ease of comparison of unit cost components shown in Fig 3 in the main text and to demonstrate the effect of PoC testing volume on the cost per test, we grouped sites according to median testing volume. Based on the distribution of PoC tests per International Organization for Standardization (ISO) week [3] across health facilities, excluding the first six weeks of study recruitment and recruitment pauses due to COVID-19 lockdowns from 13 May to 27 July 2020 in Mozambique and from 17 April to 25 May 2020 in Tanzania, we split sites into low (<5 tests per week), medium (5-12 tests per week), and high (>12 tests per week) volume categories. Median volumes per site and category divisions are shown in Fig **D**. PoC analyzers were shared with maternal viral load testing at delivery in the study. In addition, non-study related testing volume (e.g., routine HIV viral load monitoring or tuberculosis diagnosis) accounted for 12% of total testing volume in Tanzania. In Mozambique, PoC analyzers were exclusively used for study purposes.

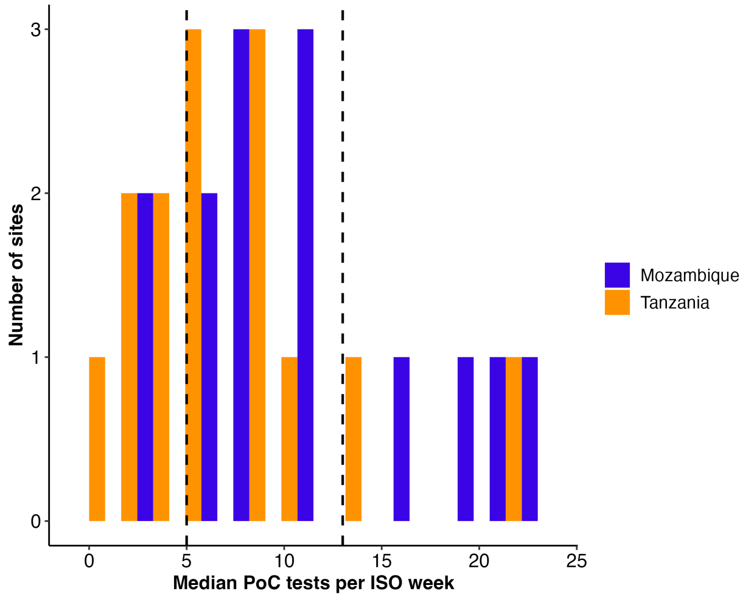

Fig D: Histogram of median PoC tests per ISO week with divisions for low-, medium-, and high-volume sites shown by dashed lines. *PoC = point-of-care; ISO = International Organization for Standardization.*

4 Supplementary methods: costing

4.1 Currency conversion rates

Cost data was collected in 2020 and 2021 and converted to United States dollars (US$) using the World Bank Global Economic Monitor "Exchange rate, new LCU per USD, period average" for the respective year [4]. At the time of the study, these were 66.8 Mozambique Metical and 2304.4 Tanzanian Shillings per 1 US$ in 2020 and 64.4 Mozambique Metical per 1 US$ for 2021. No costs paid in Tanzanian Shillings were collected for 2021. Because the analysis focuses primarily on 2020, all costs are reported in nominal 2020 US$. Inflation adjustment across years was not performed as it would have negligible impact on the results given the short time frame and single-year focus.

4.2 Capital costs

PoC platform cost was obtained from the Clinton Health Access Initiative (CHAI) and purchase invoices and included initial purchase, shipping, custom clearance, installation, insurance, and yearly maintenance. The costs

of training in the use of the PoC platforms, performed centrally per country for all study sites, were averaged between sites and did not take into account the number of trained personnel. We averaged the total cost of facility upgrades, including the purchase of cabinets to store reagents and the installation of air conditioning units in some healthcare facilities, since they were only available as aggregated costs per country. Training and facility upgrade costs were obtained from CHAI, and calculated assuming 5- and 10-year lifespans, respectively.

4.3 Discounting & Amortization

Capital costs C with value that extended beyond the study period (equipment, training, and facility upgrades) were discounted at 3% per year in the base case and amortized using an equivalent annual cost method following standard guidance [5] using

$$C(i,n) = \sum_{t=0}^{n} \frac{C_i}{(1+r)^t} \tag{1}$$

and

$$C_{yr}(i,n) = C(i,n) \frac{r}{(1-(1+r)^{-n})}, \tag{2}$$

where i denotes the type of capital cost, n represents the lifespan of the resource, and r is the discount rate per year. In the base case, we used 5-year lifespans for PoC platforms and training, and a 10-year lifespan for facility upgrades.

4.4 Overhead costs

Overhead costs, including electricity and communications (e.g., calling clients to remind them of missed appointments, communicate test results, etc.) were obtained from the Ministry of Health (MoH) in each country as an average for all study sites. In the absence of additional information, 10% of these costs were allocated to PoC EID testing. Costs for building space were not available and therefore were not included. In most sites, existing, shared space was used for interventions and upgraded to support intervention activities (see Section 4.2).

4.5 Fixed cost calculation

In the LIFE study, each site was equipped with one PoC platform, except in Mozambique where VEID sites had two. Fixed costs per site were calculated by adding annualized equipment costs (including the cost of PoC platform purchase, installation, maintenance, training, and facility upgrades) and overhead costs and dividing by the number of tests run. The fixed cost per test $C_{f,j}$ is given by

$$C_{f,j} = \frac{C_p \times N_p + C_t + C_u + C_o}{\lfloor N_j \rfloor} \tag{3}$$

where:

- C_p is the discounted annual platform cost,
- N_p is the number of PoC platforms,
- C_t is the discounted annual training cost,
- C_u is the discounted annual facility upgrade cost,
- C_o is the annual overhead cost,
- N_j is the number of PoC tests run per year, and
- $\lfloor . \rfloor$ represents the floor function.

Fixed costs were shared with routine services including HIV viral load monitoring in the study and in Tanzania, a minimal amount of non-study usage. Shared resources (including analyzer purchase, installation, maintenance, and training; electricity, and communication costs) were allocated proportionally based on PoC EID testing volume relative to total analyzer use. A 12-week EID test was for study purposes and is not part of routine guidelines. However, if infants missed a 4-8-week test but returned for the 12-week study visit, we included it in the analysis assuming that testing would occur upon return to the healthcare facility in routine practice.

For modeling analyses, annual site-level fixed costs were allocated to weeks based on the number of PoC tests performed in each week. This approach allows weekly cost estimates to reflect variation in testing throughput, while aggregate estimates converge to the annual fixed cost when averaged over time.

4.6 Variable costs

Prices for test consumables, including the test cartridge and sample collection materials, were sourced from CHAI. Salary data was obtained from MoH salary scales, with the proportion attributable to PoC EID calculated based on a 248-day work year and 38-hour work week. Nurses were asked to consider pre-test counseling, sample collection, running the PoC EID test, communication of results and post-test counseling (including counseling of families with an infant diagnosed with HIV and instruction on treatment administration if needed) in their time estimates for PoC EID. Nurses reported that it took 10 minutes on average to collect and prepare samples for PoC testing and communicate HIV-negative results and 30 minutes on average for HIV-positive results.

4.7 Variable cost calculation

The variable cost per test $C_{v,j}$ was calculated by summing the cost of consumables $C_{c,j}$ and labor costs. Labor costs were calculated by adjusting the nurse's salary S for the active work time T required for the test process (i.e., not including the time when the test was running and other tasks could be completed in parallel)

$$C_{v,j} = C_{c,j} + (S \times T). \tag{4}$$

4.8 Unit cost calculations

The unit cost per test C_j and per infant C_k were calculated as follows:

$$C_j = C_{f,j} + C_{v,j} \tag{5}$$

$$C_k = (C_{f,j} + C_{v,j}) \times N_{jk} + C_{ART,1} \times \begin{Bmatrix} 4 & Moz \\ 6 & Tan \end{Bmatrix} \times HIV_{pos}, \tag{6}$$

where $C_{f,j}$ represents the fixed costs per test, $C_{v,j}$ the variable costs per test, N_{jk} is the number of tests run per infant, $C_{ART,1}$ is the one-week antiretroviral treatment (ART) costs per infant, and HIV_{pos} is an indicator variable equal to one if the infant is diagnosed with HIV and zero otherwise.

ART costs were estimated conservatively as the minimum additional time on ART under VEID, as stipulated by study visit schedules (which followed national program visit schedules): four weeks in Mozambique and six weeks in Tanzania. By limiting the cost window to this period, we capture the specific incremental cost of ”very early” ART initiation, i.e., the 4-6 weeks of ART that the intervention group receives treatment while the SoC group is still awaiting their first test. While this approach excludes long-term ART costs, it also excludes the potential high-cost clinical complications (e.g., hospitalizations) associated with delayed diagnosis in the SoC arm, which fall outside the scope of the study. Cost estimates for central laboratory-based testing for resolution of discrepant results or in case reagents were not available at the sites were not available and therefore were not included. However, these cases represented only 1.3% of all EID tests in the LIFE study and costs are typically less than PoC EID testing costs [6].

The descriptive unit costs per country and study arm presented in the main text were summarized as testing-volume weighted means. Uncertainty was estimated using a site-level cluster bootstrap (10,000 resamples, 95% confidence interval).

5 Supplementary methods: economic model

5.1 Model overview

We utilized brms [7] to model costs and effectiveness outcomes in parallel using Bayesian hierarchical models to account for skewed cost data and clustering at the site level, while allowing differences in effectiveness associated with more frequent testing (i.e., testing at birth plus 4-8 weeks) to be captured through study arm and covariates [8]. The model was parameterized using individual-level cost and health outcome data for 6602 infants from the LIFE study. Outcomes were modeled as 1) incremental cost per additional infant initiating ART in the first week of life and 2) incremental cost per additional infant tested in the first eight weeks of life.

5.2 Cost model

For each infant k in site s, cost was estimated using a hurdle Gamma model with: 1) a zero-inflation component accounting for the presence of observed zeros in the data (i.e., SoC infants who never received an EID test due to death or loss to follow-up), modeled as the probability of cost C_{ks} being zero with $logit(C_{ks}) \sim Bernoulli(\pi_{ks})$ (Equation 7a) and 2) non-zero values modeled using a Gamma distribution $C_{ks}|(C_{ks} > 0) \sim \text{Gamma}(k_{ks}, \theta_{ks})$ (Equation 7b). The interaction of study arm T_{ks} and the total number of EID tests run per infant EID_{ks} is included as a fixed effect to reflect the fact that SoC sites had higher cost per test due to lower testing volumes at the site level. A random intercept u_s for healthcare facility nested in country and a random slope v_s for EID_{ks} are assumed to follow a normal distribution with mean zero and standard deviations τ_1^2 and τ_2^2

$$\text{logit}(P(C_{ks} > 0)) = \beta_0 + \beta_1 T_{ks} + \beta_2 EID_{ks} + \beta_3 (T_{ks} \cdot EID_{ks}) + u_s \qquad u_s \sim Normal(0, \tau_1^2) \tag{7a}$$

$$\log(C_{ks} | (C_{ks} > 0) = \gamma_0 + \gamma_1 T_{ks} + \gamma_2 EID_{ks} + \gamma_3 (T_{ks} \cdot EID_{ks}) + u_s + v_s \qquad \begin{matrix} u_s \sim Normal(0, \tau_1^2) \\ v_s \sim Normal(0, \tau_2^2). \end{matrix} \tag{7b}$$

A hurdle Gamma specification was selected over alternative models (e.g., log-Normal or zero-inflated Gamma) because, from a provider perspective, the zeros in this study represent structural non-utilization outcomes rather than latent or missing sampling zeros; specifically, infants who did not receive EID testing at the study sites due to death or loss to follow-up between birth and study follow-up. Furthermore, a Gamma distribution with a log-link was chosen over a log-Normal specification because it avoids over-sensitivity to extreme outliers and back-transformation bias often found with Log-Normal OLS models. The appropriateness of this specification was confirmed via posterior predictive checks (Fig **F**).

Priors for the standard deviation of u_s and v_s across healthcare facilities were set to reflect moderate, non-zero variability across healthcare facilities based on cost variability at the site level due to testing volume.

5.3 Effect models

The primary effectiveness outcome was defined as infants with HIV on ART in the first week of life modeled as $E_{ks} \sim Bernoulli(\pi_{ks})$ with fixed effects for study arm T_{ks}, an indicator for whether the infant had an HIV-positive test result at birth and was therefore eligible for early ART P_{ks}, and country Y_{ks} (Equation 8a. A secondary outcome was defined as infants receiving an EID test within the first eight weeks of life also modeled similarly with a fixed effect for T_{ks} and a random intercept for healthcare facility nested in country w_k, which was assumed to be normally distributed with standard deviation σ^2 (Equation 8b)

$$\text{logit}(P(E_{ks} = 1)) = \delta_0 + \delta_1 T_{ks} + \delta_2 P_{ks} + \delta_3 Y_{ks}, \tag{8a}$$

$$\text{logit}(P(E_{ks} = 1)) = \varepsilon_0 + \varepsilon_1 T_{ks} + w_s \qquad w_s \sim Normal(0, \sigma^2). \tag{8b}$$

To reflect our strong expectation of high probabilities of 1-week ART initiation and 8-week PoC EID uptake in VEID and low probabilities in SoC due to the study design, we set normal priors for the effect of δ_1 and ε_1 on E_{ks}. Means of -5 and 5 translate to, for example, probabilities of approximately 0.67% and 99% in SoC and VEID, respectively.

5.4 Parameter estimation

We performed parameter sampling for each model on four Markov chains with 8000 iterations per chain, with the first 5500 iterations discarded as burn-in. Total post burn-in draws were 10,000 for each model. Adapt delta was set to 0.98 and maximum tree depth to 14.

$\hat{R}$ statistics were all 1.00 and visual examination of parameter traces after burn-in showed acceptable mixing across chains (Fig **E**). Posterior predictive checks are shown in Fig **F**.

Intra-cluster correlation was 0.033 (95% CrI: 0.018–0.059) for cost per infant and 0.140 (0.066–0.258) for 8-week EID uptake. There were no random effects included in the 1-week ART initiation outcome model.

Table **D** provides a summary of parameters, prior distributions, and posterior means with 95% credible intervals. Posterior sampling distributions and scatter plots are shown in Figs **G** and **H**.

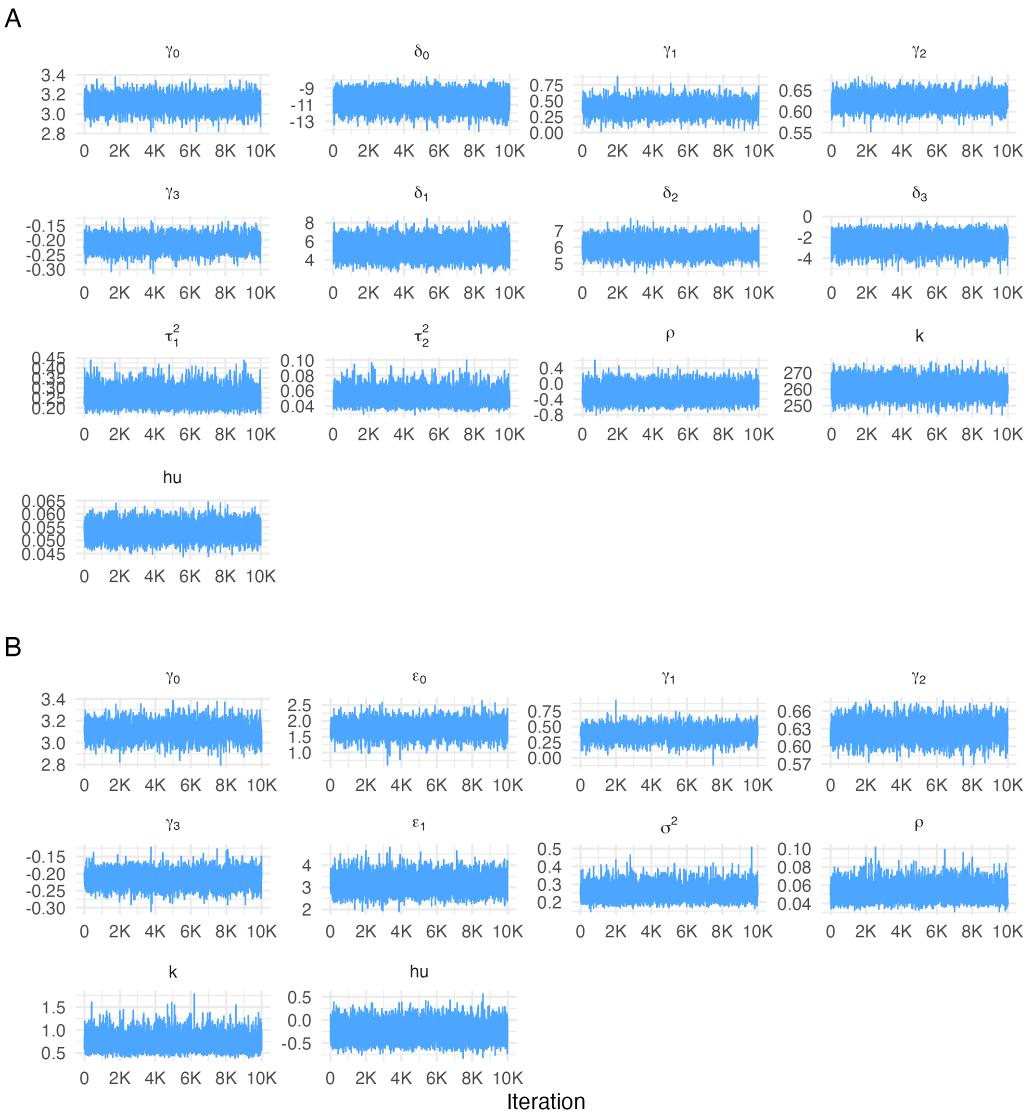

Fig E: Model parameter traces for A) 1-week ART uptake model and B) 8-week PoC EID uptake model. Burn-in phase not included. Parameters defined in Table **D**. *ART = antiretroviral treatment; PoC EID = point-of-care early infant (HIV) diagnosis.*

Param.	Component	Description	Prior	Posterior Mean (95% CrI)	Scale
β_0	Hurdle (Binary Cost)	Intercept cost probability	..	3.11 (2.97, 3.24)	logit
β_1	Hurdle (Binary Cost)	Study arm effect on cost probability	..	0.39 (0.20, 0.58)	logit
β_2	Hurdle (Binary Cost)	EID tests effect on cost probability	..	0.62 (0.59, 0.65)	logit
β_3	Hurdle (Binary Cost)	Interaction study arm and EID tests	..	-0.21 (-0.25, -0.17)	logit
γ_0	Gamma (Contin. Cost)	Intercept cost	..	3.11 (2.97, 3.24)	log
γ_1	Gamma (Contin. Cost)	Study arm effect on cost	..	0.39 (0.20, 0.58)	log
γ_2	Gamma (Contin. Cost)	EID tests effect on cost	..	0.62 (0.59, 0.65)	log
γ_3	Gamma (Contin. Cost)	Interaction study arm and EID tests	..	-0.21 (-0.25, -0.17)	log
δ_0	Bernoulli (1-wk. ART)	Intercept 1-week ART uptake	Normal(-5, 1)	-10.5 (-12.3, -8.85)	logit
δ_1	Bernoulli (1-wk. ART)	Study arm effect on 1-week ART uptake	Normal(5, 1)	5.23 (3.65, 6.92)	logit
δ_2	Bernoulli (1-wk. ART)	Positive effect on 1-week ART uptake	..	6.01 (5.19, 6.87)	logit
δ_3	Bernoulli (1-wk. ART)	Country effect on 1-week ART uptake	..	-1.78 (-2.79, -0.87)	logit
ε_0	Bernoulli (8-wk. EID)	Intercept 8-week PoC EID test	..	1.76 (1.36, 2.17)	logit
ε_1	Bernoulli (8-wk. EID)	Study arm effect on 8-week PoC EID test	Normal(5, 1)	3.15 (2.51, 3.90)	logit
τ_1^2	Random Intercept Variance	Cluster intercept variance for cost	Exponential(2)	0.25 (0.19, 0.34)	log
τ_2^2	Random Slope Variance	Cluster slope variance for EID tests	Exponential(2)	0.05 (0.04, 0.07)	log
σ^2	Random Intercept Variance	Cluster intercept variance for PoC EID	..	0.72 (0.48, 1.07)	log
ρ	Random effects	Correlation of random effects	..	-0.25 (-0.58, 0.13)	log

Table D: Summary of model parameters and posterior estimates.

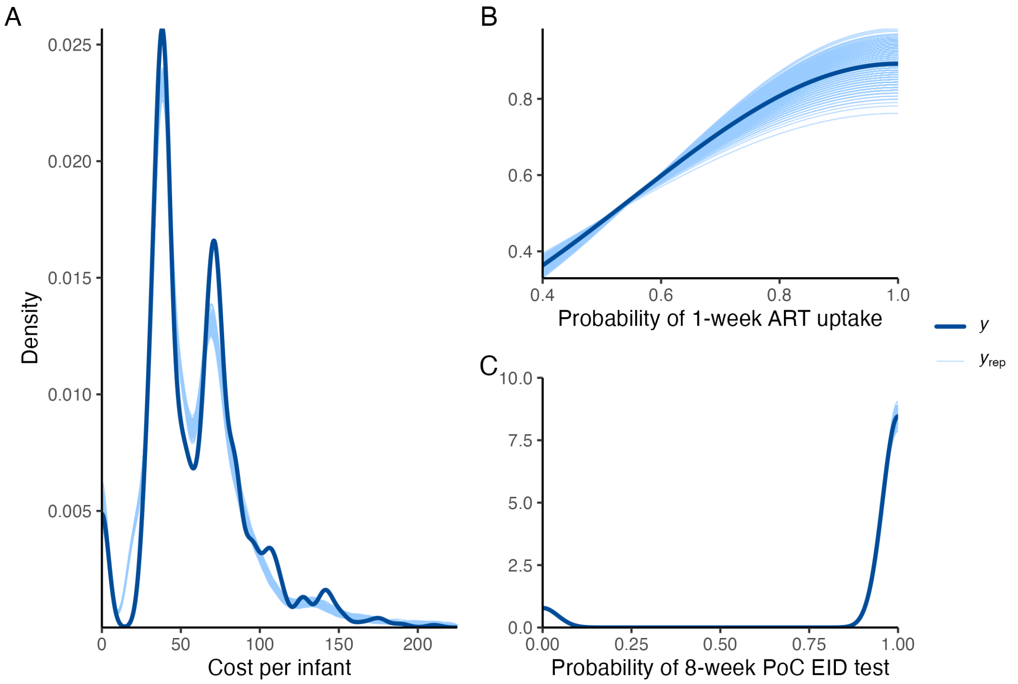

Fig F: Posterior predictive plots for A) Cost, B) 1-week ART initiation and C) 8-week PoC EID uptake. y refers to the observed data, y_{rep} to the simulated data from the posterior predictions. *ART = antiretroviral treatment; PoC EID = point-of-care early infant (HIV) diagnosis.*

5.5 Prior sensitivity

We conducted a sensitivity analysis to evaluate the impact of our prior choices on the estimated probabilities of 1-week ART initiation. Informative priors reflecting the study design were replaced with weakly informative Normal(0, 2.5) distributions to reduce prior influence. The results are shown in Table **E**.

**Param.**	**Primary Prior**	**Sensitivity Prior**	**Primary Posterior Mean (95% CrI)**	**Sensitivity Posterior Mean (95% CrI)**
δ_0	Normal(-5, 1)	Normal(0, 2.5)	-10.5 (-12.3, -8.85)	-13.1 (-15.5, -11.0)
δ_1	Normal(5, 1)	Normal(0, 2.5)	5.23 (3.65, 6.92)	5.59 (4.01, 7.22)
δ_2	..	..	6.01 (5.19, 6.87)	9.46 (7.94, 11.29)
δ_3	..	..	-1.78 (-2.79, -0.87)	-1.22 (-2.89, 0.43)
ε_0	Normal(-5, 1)	Normal(0, 2.5)	1.76 (1.36, 2.17)	1.76 (1.35, 2.14)
ε_1	Normal(5, 1)	Normal(0, 2.5)	3.15 (2.51, 3.90)	3.14 (2.51, 3.86)

Table E: Posterior estimates for primary informative directional and sensitivity weak neutral prior specifications. Parameter definitions can be found in Table **D**.

Prior sensitivity analysis indicates minimal changes to the estimated study arm effects δ_1 and ε_1, indicating that primary inferences were driven by the data rather than prior assumptions. The effect of a positive HIV test at birth δ_2 absorbed additional signal for infants with HIV initiating ART when baseline and study arm priors were weak, resulting in the observed increase for this parameter.

5.6 Intrauterine transmission probability

Since infants diagnosed with HIV at birth represented a small proportion of infants in the LIFE study, we generated distributions of probabilities for intrauterine transmission (PoC EID or retrospective dried blood spot sample positive at birth) for each country and study arm using a Bayesian bootstrap. By resampling 10,000 times from the observed data with weights w_k drawn from a Dirichlet distribution with parameters equal to one,

$$\hat{p}\text{bootstrap} = \sum k = 1^n w_k \times \tag{9}$$

we incorporated uncertainty into our estimates of test positivity at birth at the country and study arm level. Costs were then rescaled to infants with HIV using draws from the bootstrap distribution and used in subsequent analyses of outcomes related to this cohort. The resulting distributions are shown in Fig **I**.

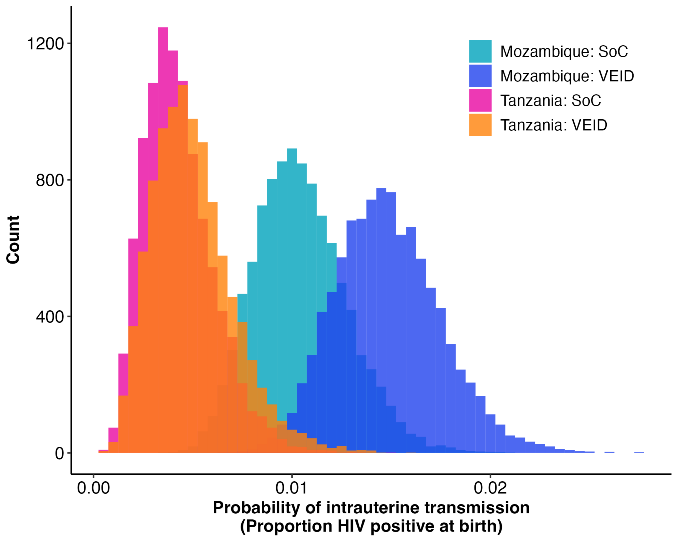

Fig I: Distributions of intrauterine transmission probabilities (defined as probability of positive HIV test result at birth) generated from 10,000 Bayesian bootstrap samples per country and study arm. *SoC = standard of care; VEID = very early infant (HIV) diagnosis.*

5.7 Incremental costs and cost-effectiveness ratios

Incremental costs, incremental effectiveness, and incremental cost-effectiveness ratios (ICERs) were calculated using the *hesim* package [9]. We provided country- and study-arm-specific posterior draws of incremental costs

and incremental effectiveness to *hesim*, which calculates ICERs as the ratio of mean incremental costs to mean incremental effectiveness $\Delta C/\Delta E$. For the 1-week ART uptake outcome, costs of HIV testing among all infants exposed to HIV were redistributed to infants diagnosed with HIV. We used draws from the intrauterine transmission probability bootstrap estimates described above per study arm and country to estimate the share of costs for each infant with HIV. In sensitivity analyses, we redistributed costs across a 0-40% intrauterine transmission rate to assess how cost-effectiveness would vary across epidemiological contexts and to identify threshold levels of transmission at which VEID would meet common cost-effectiveness benchmarks. The ICER with respect to additional infants receiving a PoC EID test within eight weeks of life were calculated with original cost per infant (i.e., without redistributing costs).

5.8 Decision analysis

We compared ICERs to empirically derived country-specific cost-effectiveness thresholds per life-year gained from [10] converted into 2020 US$ and willingness to pay (WTP) values equivalent to per capita gross domestic product (GDP) in 2020 (current US$) [4] per disablity-adjusted life-year averted. Cost-effectiveness thresholds were $189 and $316 for Mozambique and Tanzania, respectively. 1x GDP per capita was $462.40 and $1117.40, respectively. Although GDP-based WTP values are typically defined per disability-adjusted life-year averted, we used them as a contextual reference for life-years gained.

To estimate the life-years gained required to reach cost-effectiveness given typical willingness to pay values [4] [10], we used

$$LY_t = \frac{\Delta C_k}{\lambda \Delta E_{ks}}, \tag{10}$$

where LY_t is the required life-years gained per additional infant initiating ART within one week of life, ΔC_k is the incremental cost of VEID per infant with HIV, λ is the WTP threshold (US$ per life-year gained), and ΔE_{ks} is the incremental probability of ART initiation within one week of life attributable to VEID.

5.9 CHER trial benchmark

To benchmark our estimated life-years that would be needed per additional infant initiating ART within one week of life for VEID to be cost-effective, we projected the potential survival benefit of early ART initiation using outcomes from the CHER trial [11] from South Africa. The CHER trial reported lower death rates among infants who initiated early ART at 6 weeks compared to deferred ART at a median of 7 months of age over 4.8 years of follow-up. We translated the observed death counts and follow-up time per arm in CHER into estimated life-years gained per infant, propagating uncertainty using Poisson-based sampling. Early versus deferred ART was associated with 0.788 (95% CrI: 0.313–1.260) life-years gained. This projection provides an external benchmark of the potential life-years gained per infant, but we caution that differences in ART initiation timing and study contexts (e.g., adherence) may influence effect size.

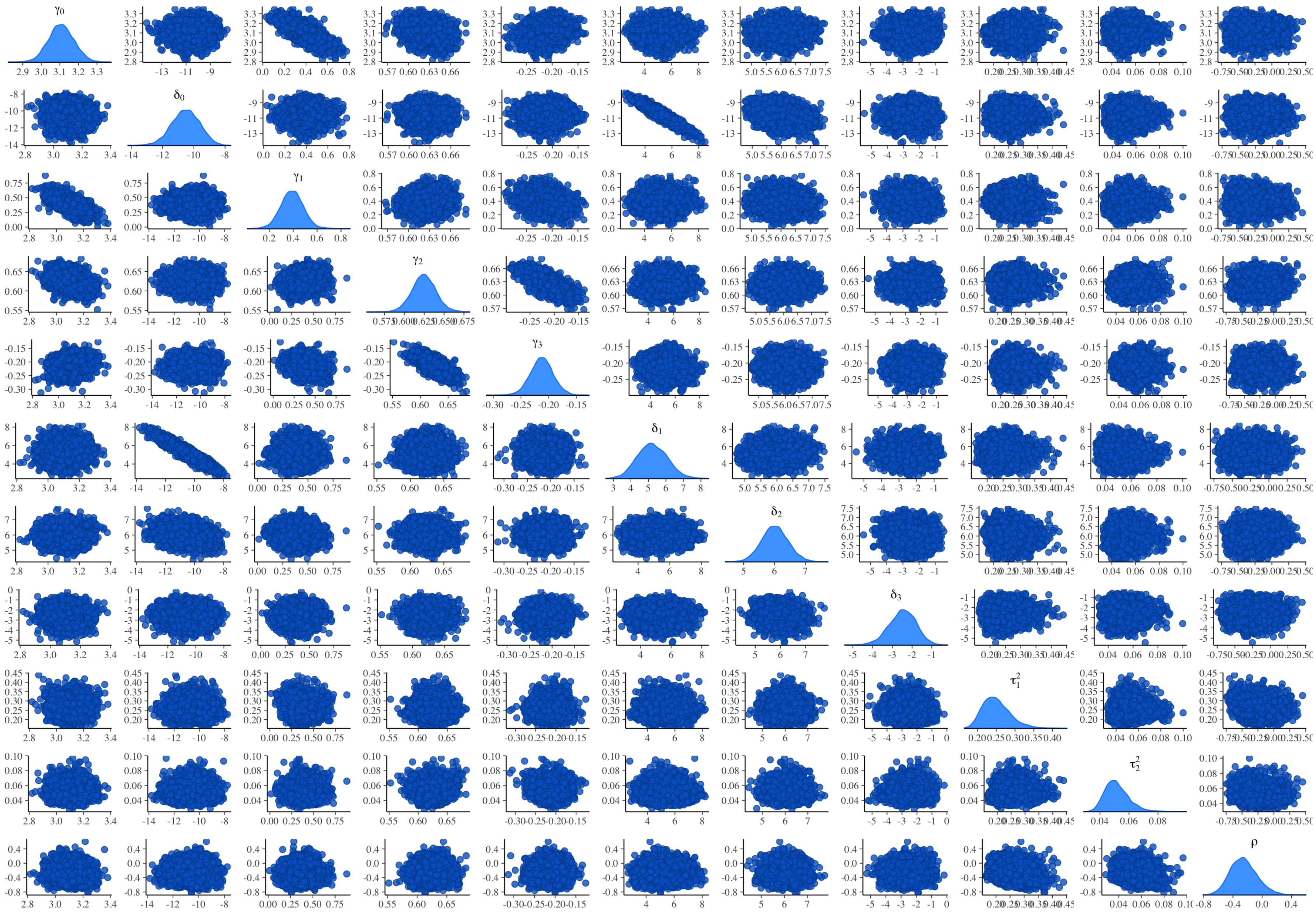

Fig G: Posterior distributions and scatter plots for 1-week ART uptake model. *ART = antiretroviral treatment.*

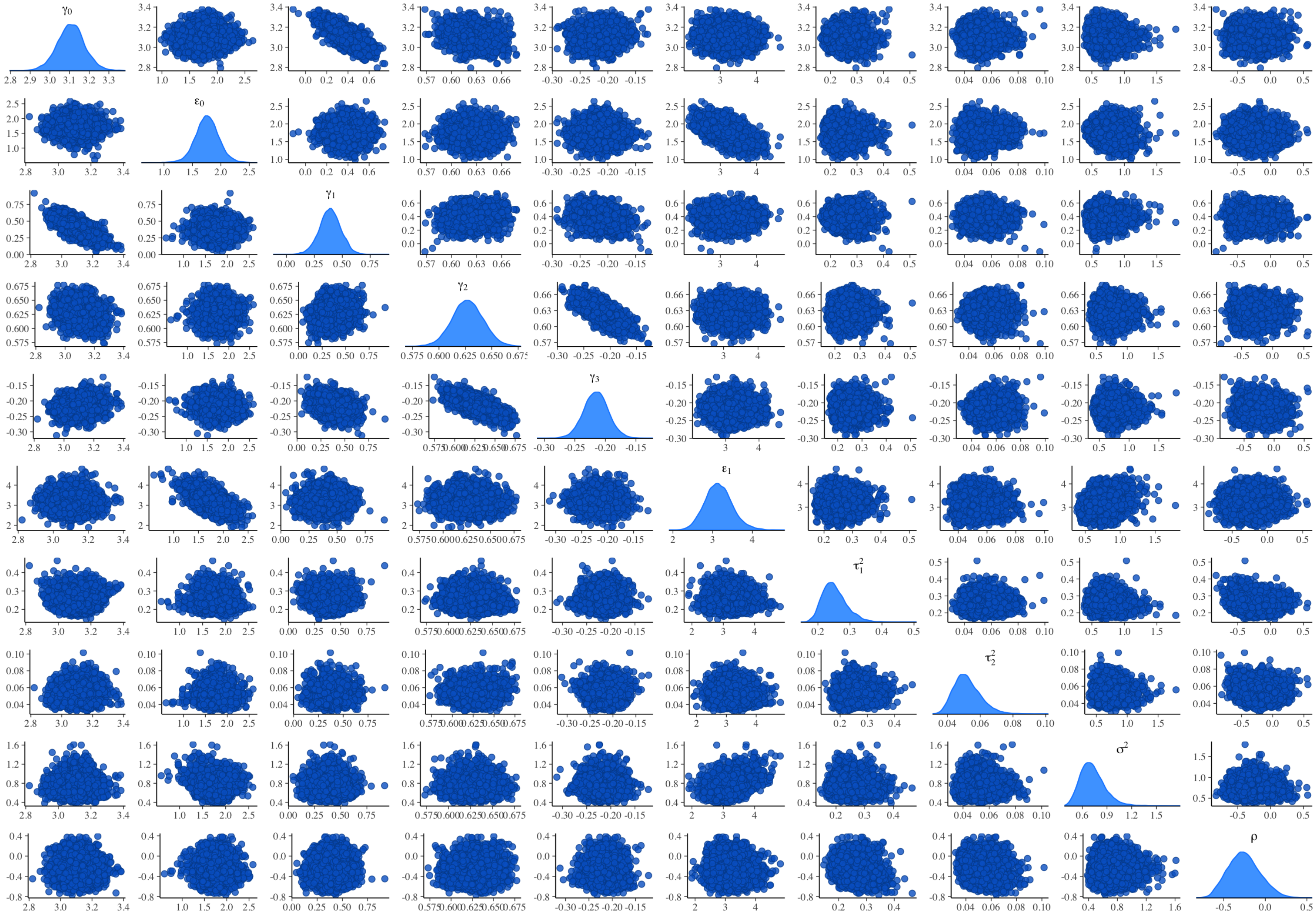

Fig H: Posterior distributions and scatter plots for 8-week PoC EID uptake model. *PoC EID = point-of-care early infant (HIV) diagnosis.*

References

[1] World Health Organization, *Who prequalification of in vitro diagnostics, product: M-pima hiv-1/2 detect assay*, 2016. [Online]. Available: https://extranet.who.int/prequal/sites/default/files/whopr_files/m-pima-hiv12-detect-pqdx-0226-032-00-public-report-v-11.0.pdf.

[2] World Health Organization, *Who prequalificaion of in vitro diagnostics, product: Xpert® hiv-1 qual assay*, 2016. [Online]. Available: https://extranet.who.int/prequal/sites/default/files/whopr_files/PQDx_0259-070-00_Xpert_HIV-QualAssay_v6.0.pdf.

[3] *International organization for standarization iso*, 2023. [Online]. Available: https://www.iso.org/home.html.

[4] *Global economic monitor (gem)*, 2021. [Online]. Available: https://databank.worldbank.org/source/global-economic-monitor-(gem).

[5] M. F. Drummond, M. J. Sculpher, K. Claxton, G. L. Stoddart, and G. W. Torrance, *Methods for the Economic Evaluation of Health Care Programmes*, Fourth. Oxford University Press, 2015.

[6] K. Elsbernd, K. M. F. Emmert-Fees, A. Erbe, *et al.*, "Costs and cost-effectiveness of HIV early infant diagnosis in low- and middle-income countries: A scoping review," *Infectious Diseases of Poverty*, vol. 11, no. 1, p. 82, 2022. DOI: 10.1186/s40249-022-01006-7.

[7] P.-C. Bürkner, "Brms: An r package for bayesian multilevel models using stan," *Journal of Statistical Software*, vol. 80, pp. 1–28, 2017. DOI: 10.18637/jss.v080.i01. [Online]. Available: https://www.jstatsoft.org/index.php/jss/article/view/v080i01.

[8] M. El Alili, J. M. van Dongen, J. L. Esser, M. W. Heymans, van Tulder M. W., and J. E. Bosmans, "A scoping review of statistical methods for trial-based economic evaluations: The current state of play," *Health Economics*, 2022. DOI: 10.1002/hec.4603.

[9] D. Incerti and J. P. Jansen, *Hesim: Health economic simulation modeling and decision analysis*, 2021. [Online]. Available: https://hesim-dev.github.io/hesim/.

[10] A. Pichon-Riviere, M. Drummond, A. Palacios, S. García-Martí, and F. Augustovski, "Determining the efficiency path to universal health coverage: Cost-effectiveness thresholds for 174 countries based on growth in life expectancy and health expenditures," *The Lancet Global Health*, vol. 11, no. 6, e833–e842, 2023. DOI: 10.1016/S2214-109X(23)00162-6.

[11] M. F. Cotton, A. Violari, K. Otwombe, *et al.*, "Early time-limited antiretroviral therapy versus deferred therapy in south african infants infected with hiv: Results from the children with hiv early antiretroviral (cher) randomised trial," *Lancet*, vol. 382, no. 9904, pp. 1555–1563, Nov. 2013. DOI: 10.1016/S0140-6736(13)61409-9.
