# Supplementary material for: Point-of-care early infant HIV diagnosis at birth in a pragmatic cluster-randomized trial in Mozambique and Tanzania: A comparative cost and cost-effectiveness study
Source: PLoS Med. 2026 May 6;23(5):e1005069. doi: 10.1371/journal.pmed.1005069 (PMC13166934; doi:10.1371/journal.pmed.1005069)
Supplement: Attachment — Submitted filename: Review_response_PlosMed.pdf [file pmed.1005069.s005.pdf]

Author's responses to editors' and reviewers' comments for manuscript PMEDICINE-D-25-00626R1

Comparative cost and cost-effectiveness of point-of-care early infant HIV diagnosis at birth: Findings from a pragmatic cluster-randomized trial in Mozambique and Tanzania

Kira Elsbernd, Issa Sabi, Ilesh Jani, Chishamiso Mudenyanga, Siriel Boniface, Arlete Mahumane, et al.

Note: Editors' and Reviewers' comments are typeset in grey boxes and changes to the manuscript and supplement are typeset in yellow boxes. Rephrased or added text is typeset in blue. The respective changes in the tracked changes version of the manuscript are highlighted to indicate changes.

Note: In reviewing the comments, we noticed an error in the discount rate used in some calculations. The corrected numbers have been updated in the abstract, Table 1, and in the descriptive results of costs under "Costs of very early infant diagnosis versus standard-of-care." These corrections do not affect the overall conclusions of the manuscript.

Response to the Academic Editor

In the current global funding climate and uncertainties about PEPFAR funding continuation it is likely that rate of vertical transmission will go up again, given that ART provision and adherence over pregnancy is likely to be affected negatively. Very early infant diagnosis would thus become more important still.

Further, the diagnosis of non-infection at birth would identify potential transmission risk through breastfeeding (which is now uncommon among infected mothers provided they are on ART), and again this risk may increase if women postpartum are unable to access ART reliably. These issues, should be mentioned in the discussion with an indication that the landscape of vertical transmission is likely to change if PEPFAR funding is withdrawn, in which case identifying infants most at risk is important.

This randomised trial did not compare birth with 6 weeks diagnosis, and only studies whether the birth test adds to the standard of care 6 weeks test. This should be discussed.

Thank you for these important comments. We agree that substantial reductions in international financial support for HIV, particularly uncertainties around PEPFAR, are likely to increase vertical transmission. A recent modeling study supports this concern, showing that new HIV acquisitions among children could increase roughly 3-fold [1].

In these scenarios, HIV prevention and testing (e.g., PrEP, routine HIV testing during antenatal care and maternal repeat testing at delivery/postpartum) are more likely to be disrupted first, while ART is often prioritized. Depending on where services erode in the prevention of vertical transmission cascade, both in utero and breastfeeding transmission could increase.

Our study was designed to evaluate whether the addition of birth testing provides immediate incremental benefit beyond the current standard of care of testing only at 4-8 weeks. This is in line with WHO guidelines which do not recommend replacing 4-8 week testing but rather adding birth testing alongside already strong 4-8 week testing programs. Our analysis focuses on HIV acquired in utero, as these are the cases where birth testing (and immediate ART initiation) can have an impact. Although identifying infants at risk of breastfeeding transmission is critical, it is not necessarily influenced by knowing the HIV negative status of an infant at birth, since this risk is present whether an HIV status at birth is known or not. Breastfeeding transmission is beyond the scope of this study.

We have added the following text to the discussion to highlight these points and emphasize that both impact and cost-effectiveness of birth testing become greater with increased in utero transmission.

Original submission
Focusing on primary healthcare settings in Mozambique and Tanzania, this study estimated the cost and cost-effectiveness of offering PoC HIV diagnostic services at birth and 4-8 weeks of age compared to the SoC at 4-8 weeks only. As vertical transmission rates continue to decline and the number of neonates benefiting from very early ART is shrinking in many sub-Saharan African settings, this information is crucial for guiding investment and design decisions in EID programs.

Changes to the manuscript
Focusing on primary healthcare settings in Mozambique and Tanzania, this study estimated the cost and cost-effectiveness of offering PoC HIV diagnostic services at birth and 4-8 weeks of age compared to the SoC at 4-8 weeks only. In the context of uncertain global funding for HIV programs, particularly recent funding freezes and proposed cuts to the President's Emergency Plan for AIDS Relief (PEPFAR), the landscape of HIV epidemiology and vertical transmission may change substantially [25, 26]. Amid this uncertainty, generating evidence on the cost and benefits of VEID is crucial for guiding investment and design decisions in EID programs, especially as transmission rates and resource availability evolve.
...
Additionally, all infants should receive follow-up testing to identify those with undetectable virus at birth or acquiring HIV during the breastfeeding period [3,16], especially considering potential disruptions to maternal ART access due to funding cuts.
...
While we did not assess cost-effectiveness of PoC versus laboratory-based EID, our estimated costs of PoC EID were comparable to costs of laboratory-based EID tests reported in other studies [28]. We instead compared PoC birth plus 4–8 week testing with PoC 4–8 week testing alone, consistent with WHO guidelines [3]. This likely underestimated the added impact of VEID, as SoC also benefited from shorter PoC turnaround times compared with central laboratory-based testing.

Response to Reviewer 1

This is a generally well written manuscript. The intention was to undertake a comparative cost and cost-effectiveness study of a point of care early HIV diagnostic test based in two countries (Mozambique and Tanzania) based on a pragmatic cluster-randomised trial.

Thank you for your constructive comments. Our point-by-point responses are below.

Comment 1

In the introduction, there should be a brief description of why these two particular countries were chosen.

We added the following to the introduction.

This study was conducted within a pragmatic trial of PoC EID at birth in public primary healthcare facilities in Mozambique and Tanzania, where high HIV prevalence among women and persistent vertical transmission [1] suggest that adding birth test-and-treat could have a meaningful impact on HIV-related early infant mortality and morbidity.

Comment 2

In the CHEERS checklist it is stated that the HEAP is reported on page 4 of your manuscript but this is incorrect and there is not mention of a HEAP.

We added a sentence to the methods.

Analyses followed a pre-specified health economic analysis plan.

Comment 3

There should be justification for why the healthcare system perspective was chosen and why a perspective which also captures out-of-pocket payments was not.

Thank you for pointing this out. We acknowledge the importance of considering out-of-pocket expenses in economic evaluations. In our study setting however, patient costs were minimal, infrequently reported, and often took the form of non-monetary trades (e.g., barter or in-kind exchanges) which are difficult to quantify reliably. As they are unlikely to be major drivers in the overall cost, we focused our analysis on health system costs to align with budgeting priorities and to provide results most relevant for policy and resource allocation decisions.

We did collect data on out-of-pocket expenses and plan to publish these findings in a subsequent manuscript. This approach allows us to present the more focused health system cost analysis here while still addressing patient costs and context comprehensively elsewhere.

We added the following to the methods to clarify the perspective choice.

We conducted a micro-costing study from the healthcare provider perspective. We estimated implementation and operations costs as they occurred in the LIFE study, including fixed costs (equipment purchase, installation, and maintenance; utilities; communication; training; and facility upgrades) and variable costs (consumables, labor, and ART) for VEID and SoC approaches. Given that patient costs in our study were minimal and often non-monetary trades, we focused on direct health system costs which are practical to capture and reflect the expenditures most relevant for planning and resource allocation.

Comment 4

A brief description of why the Abbott and Cepheid technologies were chosen should be provided given that each country only used one of the two machines. What impact is this likely to have on the design, results and conclusions drawn.

Different technologies were used in participating countries according to preexisting preferences of the local HIV programs and decision-makers. The LIFE study, upon which this cost-effectiveness study is based, chose to align with these preferences to maximize implementation uptake and sustainability. Both platforms have similar diagnostic performance, run-time, and operational requirements for early infant diagnosis, so this choice did not have a substantive impact on study design or clinical outcomes. While there are some differences (e.g., maintenance costs, Xpert can process multiple samples in parallel, mPIMA comes with an external battery which may reduce downtime during power cuts), any operational or cost implications were accounted for by incorporating country- and platform-specific costs and repeat testing rates and country-level adjustments in the economic models. We added the following to the testing equipment section in the methods.

> The Abbott mPIMA HIV-1/2 Detect and Cepheid Xpert HIV-1 Qual were used for EID testing in Mozambique and Tanzania, respectively, reflecting preexisting local HIV program preferences.
> ...
> Both analyzers have comparable diagnostic performance and run-time for EID [19, 20]. Therefore, we expect that the choice of platform did not affect linkage to ART or other health outcomes.
> ...
> Models were fit separately for 1-week ART and 8-week PoC EID outcomes with country- and site-level adjustments to reflect differences in costs, PoC testing platforms, prophylaxis protocols, and transmission rates (further details in S1 Supplementary Methods).

Comment 5

In the CHEERS checklist it is stated that the time horizon is reported on page 4 but this is not correct. The time horizon is of particular importance given that the platforms evaluated have an assumed 5-year lifespan. What was the time horizon?

Thank you for pointing out this oversight. The primary trial was unable to show a statistically significant difference in 18-month mortality between arms due to lower than expected vertical transmission rates. Therefore, we chose to report epidemiologically-important early outcomes (*ART initiation by 1 week* as the primary effectiveness outcome and *tested by 8 weeks* as a secondary effectiveness outcome). The time horizon for individual-level costs and outcomes thus corresponds to the 12-week follow-up period. Capital costs were distributed over 5-10 years to reflect their useful life beyond the study follow-up period. We have added the following to the methods to clarify these points.

> The time horizon for individual-level costs and outcomes corresponded to the 12-week follow-up period.
> ...
> Capital costs were discounted at 3% per year and amortized using equivalent annual cost—PoC platform costs over a 5-year lifespan according to manufacturer specifications and other capital costs over 10 years...

Comment 6

At no point in the manuscript did I see any rationale as to why the study would be undertaken in these two countries using different machines, processes and underlying transmission rates.

Different technologies were used according to preexisting preferences of the local HIV programs and decision makers (see also the response to Comment 4). Similarly, testing schedules were designed around existing routine EID protocols. These choices were made to ensure alignment with established programmatic practices for maximum feasibility and sustainability beyond the study.

Selection of these two countries was based on long-standing research collaborations and strong relationships with national HIV programs and implementing partners, which were essential for successful implementation of the study interventions. Both countries have high HIV prevalence among pregnant women [2]–[4] (the target population for this intervention) and consistently rank among the top 10 globally for vertical transmission according to UNAIDS estimates [2]. Real-world data on vertical transmission during the planning of the LIFE study was scarce, particularly for rural areas where many of our study sites were located. Available data from our previous studies in this setting [5] suggested higher transmission rates than what we ultimately observed in the LIFE study.

In this manuscript, we present the empirical data from the LIFE study as observed. The aim was not to directly compare countries or PoC testing platforms, but rather to assess real-world implementation in two similar but distinct programmatic contexts without attempting to artificially standardize scenarios to make such one-to-one comparisons.

We hope that these clarifications and changes under Comments 1 and 4 adequately address these concerns.

Comment 7

Having read the manuscript I am still unsure what to do with the findings - the discussion and conclusions should be framed to allow some recommendation to be made other than more research being undertaken.

We were cautious with the interpretation in the original submission, but have added additional analyses which we hope make the practical implications of our study stronger. Specifically, we added 1) a threshold analysis of the life-years gained needed for our intervention to be considered cost-effective under conventional willingness to pay thresholds which we benchmark against 5-year mortality outcomes in the CHER trial [6] in the discussion (shown below) and 2) a sensitivity analysis to show how the ICER per 1-week ART initiation is affected by intrauterine transmission rate (full details available in response to Reviewer 2's Comment 5). We also point to our concluding paragraph which recommends that countries with low transmission rates consider targeted VEID based on risk criteria (which we are currently evaluating in a related ongoing study).

Changes to the manuscript

Methods: To predict the number of life-years that would need to be gained per additional infant initiating ART in the first week of life for VEID to be considered cost-effective, we used posterior draws of incremental cost and incremental effect from the 1-week ART initiation model to propagate parameter uncertainty reported and median and 95% CrI.

Results: At cost-effectiveness thresholds from [21], VEID would need to achieve 16.27 (95% CrI: 13.49–22.78) life-years gained per early ART initiation in Mozambique and 28.06 (18.38–83.12) in Tanzania to be cost-effective (Figure 5). At thresholds equivalent to GDP per capita, the required life-years were 6.65 (5.52–9.32) in Mozambique and 7.94 (5.20–23.51) in Tanzania.

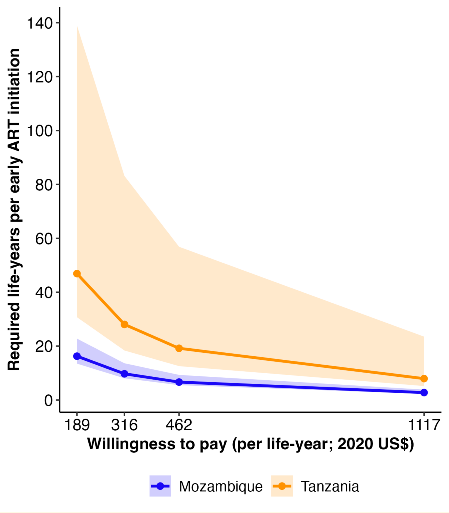

Figure 5: Required life-years per early ART initiation for cost-effectiveness. Shaded ribbons for Mozambique and Tanzania indicate 95% credible interval (CrI) across posterior draws from the Bayesian hierarchical model for 1-week ART initiation. Empirically derived cost-effectiveness thresholds, based on health expenditure and life expectancy growth [21] and expressed in 2020 US$, were $189 in Mozambique and $316 in Tanzania. Willingness to pay thresholds equivalent to per capita gross domestic product in 2020 were $462 in Mozambique and $1117 in Tanzania. Higher required life-years reflect scenarios in which greater health gains per infant would be needed to approach the indicated thresholds.

Discussion: Our analysis indicates that the survival benefits required for VEID to be considered cost-effective, 16–28 life-years per early ART initiation using cost-effectiveness thresholds from Pichon Riviere et al. [21] or 6–8 life-years using GDP-based thresholds, are substantially higher than those demonstrated to date. In the CHER trial, which remains the primary benchmark [14], early versus deferred ART was associated with roughly 0.8 life-years gained over five years, based on reported deaths and follow-up time per arm. However, CHER compared 6-week to median 7-month ART initiation, while our study compared birth to 4–8-week ART initiation. Importantly, HIV-related infant mortality peaks around 2–3 months [5], suggesting that ART at birth could be underestimated. While early mortality reductions in the LIFE study were unfortunately not sustained through 18 months, most infants received LPV/r and viral suppression was low and associated with poor clinical outcomes overall [15]. With dolutegravir-containing ART now available for infants and expected to address common barriers to adherence due to once daily dosing and improved palatability, greater longer-term health benefits may be achievable than observed in earlier cohorts. Differences in comparator, follow-up, and treatment context caution against directly extrapolating CHER outcomes to our setting and highlight that long-term benefits of VEID are still uncertain.

Conclusion: Our analysis suggests that cost-effectiveness depends on whether ART initiation at birth translates to substantial long-term health benefit or if VEID can be targeted to infants

at high risk of HIV acquisition. In settings with high vertical transmission rates or where weak HIV programs lead to significant gaps in infant retention in care, EID programs could consider adding PoC birth testing with careful consideration of PoC infrastructure utilization to improve affordability. In settings with low vertical transmission rates, the marginal benefits of universal birth testing are limited and targeted testing of high-risk infants at birth may be a more practical approach.

Response to Reviewer 2

This is an interesting paper that looks into the costs and cost-effectiveness of POC birth testing. It's an important topic, especially as national programs need to set priorities with limited resources, but could be improved by addressing a few comments.

Thank you for your thoughtful and constructive suggestions for improvement. We address each comment in the following.

Comment 1

Given this is a cost-effectiveness paper, it is unclear why DALYs weren't included as well.

Thank you for the comment. Cost-effectiveness analysis broadly compares costs with relevant outcomes and is not limited to utility-based measures such as DALYs. In this study, we focused on program-relevant outcomes (*ART initiation by 1 week* and *tested by 8 weeks*), which reflect immediate health system priorities and available data. We believe our approach provides practical insights for decision-makers regarding EID implementation.

Estimating DALYs or QALYs would require longer-term clinical and quality of life data, which were beyond the scope of this study. Furthermore, assessing quality of life in infants is particularly challenging and often not feasible. In our study, converting our observed outcomes to DALYs would require modeling and strong assumptions, whereas our focus was an empirical cost-effectiveness analysis. To connect our outcomes to conventional cost-effectivenss thresholds, we added a threshold analysis estimating the life-years gained needed for our intervention to be considered cost-effective, which we benchmarked against 5-year mortality outcomes in the CHER trial [6] (Figure 5, shown below). Full details of this analysis are presented in response to Reviewer 1's Comment 7

We also modified the limitations as follows.

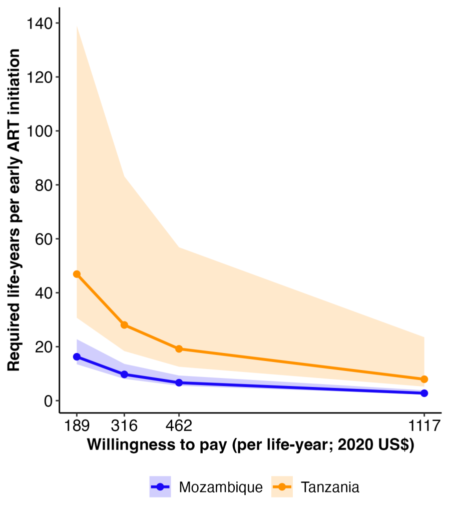

Figure 5: Required life-years per early ART initiation for cost-effectiveness. Shaded ribbons for Mozambique and Tanzania indicate 95% credible interval (CrI) across posterior draws from the Bayesian hierarchical model for 1-week ART initiation. Empirically derived cost-effectiveness thresholds, based on health expenditure and life expectancy growth [21] and expressed in 2020 US$, were $189 in Mozambique and $316 in Tanzania. Willingness to pay thresholds equivalent to per capita gross domestic product in 2020 were $462 in Mozambique and $1117 in Tanzania. Higher required life-years reflect scenarios in which greater health gains per infant would be needed to approach the indicated thresholds.

In addition, scarce long-term health data for infants with HIV—particularly survival times or quality-of-life measures—make assessing the efficiency of interventions challenging. We assessed cost-effectiveness using epidemiological outcomes relevant to EID programs as key indicators of health benefit, given the absence of significant differences in longer-term health outcomes [15], and compared ICERs to empirically derived cost-effectiveness thresholds as well as GDP per capita [21, 35, 36]... This approach excludes explicit examination of cost-utility in terms of quality- or disability-adjusted life years.

Comment 2

It would be very beneficial, given previous cost-effectiveness work on POC, and some of the discussion, for the authors to include a scenario where the devices are shared/integrated with other diseases. There are some results on this included in the discussion, but a clear analysis should be incorporated in the results.

We agree that cost-sharing of point-of-care testing infrastructure across programs is the way forward. In practice, this is not yet common and health systems in this setting remain largely siloed. In our study, PoC analyzers were shared for maternal viral load testing, and this is incorporated in the cost estimates presented. We also mention cross-utilization across programs as a means of reducing costs in the discussion. However, detailed analysis of integration scenarios, including analyzer utilization over time and cost implications, is based on additional simulations and being prepared for a separate publication. To keep the focus of this manuscript on the empirical outcomes, we did not include these simulated scenarios in the results.

To illustrate a hypothetical scenario for the discussion, we added the following.

...Without initial equipment investment (i.e., in scenarios where existing PoC testing infrastructure allows for repurposing of analyzers for PoC EID or integration with other programs), cost per test can be reduced by up to 43% for mPIMA in Mozambique and 47% for Xpert in Tanzania. However, as manufacturers specify relatively short analyzer lifespans, estimating costs with initial equipment investment for EID remains important for comprehensive budget planning. Further, cross-utilization of PoC infrastructure across programs (e.g., HIV viral load, tuberculosis) may be a viable approach to increase testing volume and decrease costs, especially at low-volume sites. At 70% utilization, cost per test was reduced by up to 4% for mPIMA in Mozambique and 27% for Xpert in Tanzania, highlighting an advantage of the Xpert platform's broad multiplex diagnostic capability. In both countries, leveraging existing PoC infrastructure and increasing PoC analyzer utilization can roughly halve costs and yield substantial efficiency gains.

Comment 3

It would be helpful to more clearly articulate why the cost of the test is lower for VEID than SOC when the same test is used, per country. Where do those "cost-savings" happen?

We added the following.

... Equipment and overhead costs, by contrast, varied substantially by testing volume. Lower cost per test for the VEID approach reflects higher testing volumes, since infants were tested twice by design and fixed costs were allocated across more tests.

Comment 4

It is clear and positive that the authors took a slightly different approach to cost-effectiveness by using the health expenditure per capita. However, either include why GDP was not selected more explicitly in the discussion and methods or include that as well.

Thank you for this comment. We clarified the choice of cost-effectiveness threshold based on Pichon-Riviere et al. [7] in the methods and added GDP per capita for comparability to the life-years gained threshold analysis. We also added the exact values used for both thresholds to the supplement.

Changes to the manuscript

We compared ICERs to empirically derived country-level cost-effectiveness thresholds from Pichon-Riviere et al. [21], which are based on health expenditure and life expectancy growth, as well as GDP per capita for comparability with previous studies.

...

At cost-effectivness thresholds from [21], VEID would need to achieve 16.27 (95% CrI: 13.49–22.78) life-years gained per early ART initiation in Mozambique and 28.06 (18.38–83.12) in Tanzania to be cost-effective (Figure 5). At WTP thresholds equivalent to GDP per capita, the required life-years were 6.65 (5.52–9.32) in Mozambique and 7.94 (5.20–23.51) in Tanzania.

Changes to the supplement

5.7 Decision analysis

We compared ICERs to empirically derived country-specific cost-effectiveness thresholds per life-year gained from [8] converted into 2020 US$ and gross domestic product (GDP) per capita in 2020 (current US$) [4]. Cost-effectiveness thresholds were $189 and $316, for Mozambique and Tanzania, respectively. 1x GDP per capita was $462.40 and $1117.40, respectively.

Comment 5

The results could be strengthened further by:

- a. The first section and Figure 2 included primary impact data that did not seem part of this cost-effectiveness work, but likely part of the original CID 2024 publication. Instead, this should be shortened or an abbreviated portion put into the methods with a supplementary table as a reference point used, not suggested as new data generated. Likewise, this should be reduced/limited in the discussion.
- b. It would be helpful to more clearly note what the costs per test were comprised of. Figure 3 helps; however, distinct dollar values for each portion and how the device was amortized would be helpful.
- c. Further, Table 1 is good, but doesn't provide a clear link to the cost per test. It would be helpful here to show how you break down each line item to contribute to the costs identified.
- d. Another sensitivity analysis that should be included would be revising the device lifespan to what both countries see as typical/median life spans for the specific devices and/or POC instruments more generally.
- e. Further, a final sensitivity analysis would be more clearly showing the transmission rates where POC birth testing becomes cost-effective.

a. The original CID 2024 publication presented impact data only for infants diagnosed with HIV, rather than for the entire cohort of infants exposed to HIV. It also reported turnaround times for testing and treatment initiation at birth, but not at later time points. Because the outcomes *tested by 8 weeks* and *ART initiation by 1 week* were key in our cost-effectiveness analysis, we considered it important to show these data here. However, we moved the transmission rate data to the methods section, slightly shortening this part of the results.

b. We revised Figure 3 and separated it into two panels: one with the dollar values (absolute picture) and one with percentages (relative picture) for consumables and equipment which make up the majority of the test cost. Overhead and labor costs were small, so we added a note to the figure legend to avoid text clutter on the figure itself. Figure S2 was revised in the same manner with costs by site. Details on the amortization method were added to the methods and supplement.

c. The intent of Table 1 was to present the input costs that were used to derive the unit costs, while Figure 3 presents the resulting unit cost per test and the relative contributions of each input category. We have clarified this link in the text by explicitly stating how equipment, facility upgrades, training, and overhead were amortized across sites and tests, and how consumables and personnel time were allocated

per test. We hope that together, Table 1 (inputs) and Figure 3 (outputs) now illustrate both the sources of costs and their contribution to the final unit costs.

d. Thank you for this comment. We replaced Table S3 (where our deterministic scenario model for per-test one-way sensitivity analysis was originally hidden away) with a tornado plot in the main text. This now visually shows how assumptions about consumable prices, testing volume, equipment lifespan, etc. influence cost per test, which we hope is easier to read. The plot is included as Figure 6.

e. We replaced Table 2 with a figure (Figure 7) to show how ICER decreases with increasing transmission rate at birth and added threshold values for transmission rate to the text.

Changes to the manuscript: Costs

Capital costs were discounted at 3% per year and amortized using equivalent annual cost—PoC platform costs over a 5-year lifespan according to manufacturer specifications and other capital costs over 10 years.

...

Cost inputs used to derive unit costs per test and per infant are listed in **Table 1**.

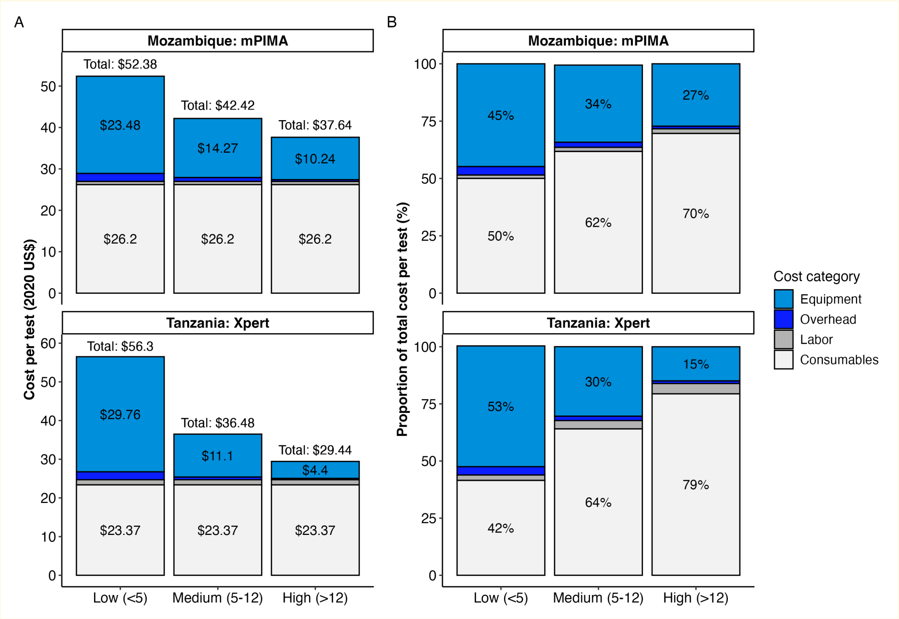

Figure 3: Point-of-care early infant diagnosis test cost components. (A) Cost estimates expressed in 2020 US$. (B) Proportion of total cost per test. Sites are categorized as high (>12 tests per week), medium (5-12 tests per week), and low (<5 tests per week) testing volume. Equipment includes amortized costs of initial purchase, installation, and yearly maintenance of PoC analyzers. Overhead includes apportioned costs of electricity and facility upgrades. Overhead and labor costs were <5% of cost per test across all sites.

Changes to the supplement: Costs

Discounting & Amortization

Capital costs (equipment, training, and facility upgrades) with value that extended beyond the study period were discounted at 3% per year in the base case and amortized using an equivalent

annual cost method...

Changes to the manuscript: Sensitivity analyses

Cost per test was most sensitive to changes in the price of consumables, followed by testing volume and PoC analyzer lifespan (**Figure 5**).

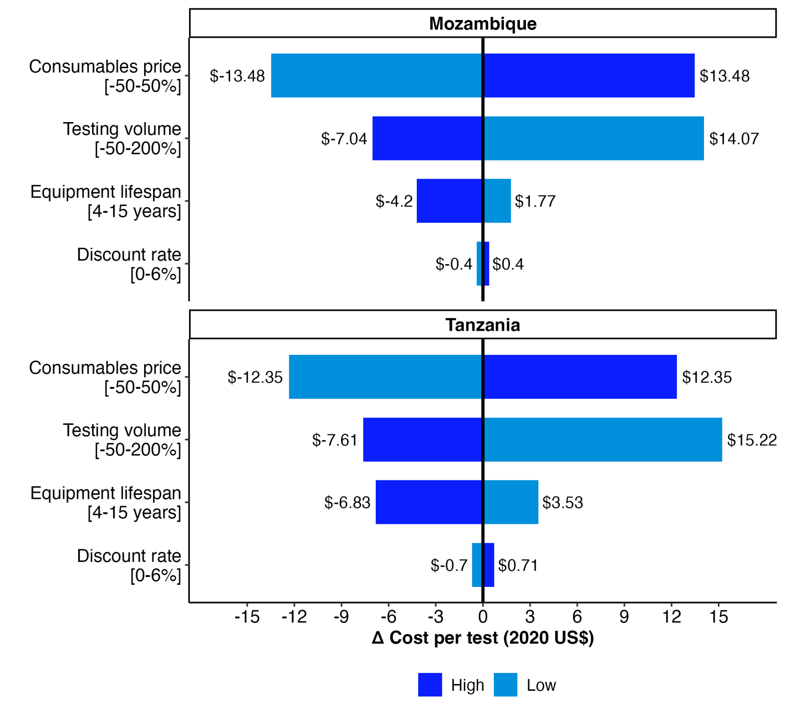

Figure 6: Deterministic one-way sensitivity analysis showing influence of key assumptions on cost per test. Costs expressed in 2020 US$. Per test cost difference is shown on the x-axis and parameters with ranges evaluated are shown on the y-axis. High and low colors indicate the direction in which the parameter varies.

In Mozambique, intrauterine transmission would need to exceed 34% to meet the cost-effectiveness threshold from [21] or 14% to meet the GDP-based threshold (**Figure 7**). In Tanzania, the corresponding intrauterine transmission rates were 20% and 5.8%, respectively, indicating cost-effectiveness is highly sensitive to the baseline risk of vertical transmission detectable at birth.

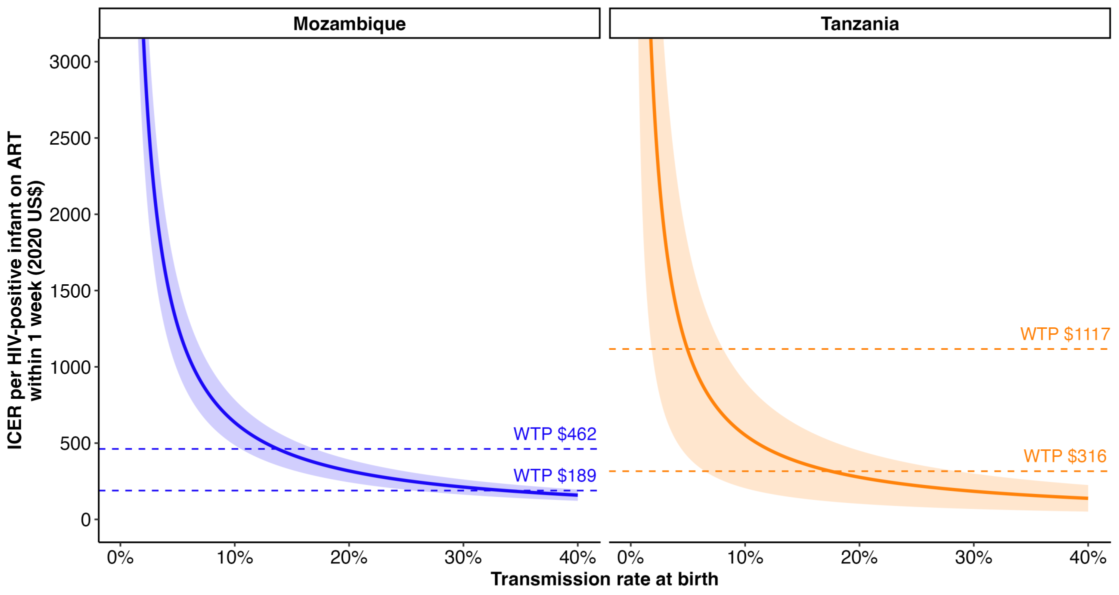

Figure 7: Sensitivity analysis of the incremental cost effectiveness ratio (ICER) per HIV-positive infant on ART within 1 week across a range of intrauterine transmission rates, based on posterior estimates from the Bayesian hierarchical model. Lines show the posterior mean ICER, with shaded areas representing the 95% credible intervals. Dashed horizontal lines indicate reference willingness to pay (WTP) thresholds: empirically derived cost-effectiveness thresholds based on health expenditure and life expectancy growth from [21], expressed in 2020 US$ ($189 for Mozambique and $316 for Tanzania), and gross domestic product per capita in 2020 ($462 for Mozambique and $1117 for Tanzania).

Comment 6

The cost per infant should be defined more clearly. In particular, highlighting that it is higher than per test and SOC because they are receiving an additional test. Why are the per infant costs of SOC different from the cost per HIV test? Aren't both only receiving one test and no different cost components are included in the former that weren't in the latter?

Although testing costs make up the majority of the cost per infant (1–2 tests per infant, depending on study arm), additional costs of earlier ART initiation are also included in the per-infant cost. In addition, cost per test varies by testing volume at the site level, as shown in Figure 3. VEID sites naturally had higher testing volumes, as they performed roughly twice as many tests. In VEID sites in Mozambique, an additional analyzer was procured to handle higher testing volumes, so the per-test cost did not decrease substantially. In contrast in Tanzania, where each site had only one analyzer, cost per test was lower in VEID sites due to higher testing volume on the same analyzer.

Changes to the manuscript
Cost per HIV-exposed infant, which incorporates PoC EID testing and if appropriate, earlier ART initiation was...

Comment 7

In the discussion, I would suggest noting that one limitation is the use of POC as SOC and that this is likely to underestimate impact when lab-based testing is used.

Thank you for this comment. We added the following to the discussion.

> While we did not assess cost-effectiveness of PoC versus laboratory-based EID, our estimated costs of PoC EID were comparable to costs of laboratory-based EID tests reported in other studies [28]. We instead compared PoC birth plus 4–8 week testing with PoC 4–8 week testing alone, consistent with WHO guidelines [3]. This likely underestimated the added impact of VEID, as SoC also benefited from shorter PoC turnaround times compared with central laboratory-based testing.

Comment 8

The authors shied away from providing a clear conclusion to their work. From what it seems, POC birth testing is not at all cost-effective. This is unfortunate, but it is what the data show. Laying that out clearly should be done, not waiting for the last paragraph. However, to this point, a positive spin could be more possible should sensitivity analyses from points 2, 5d, and 5e be completed. Then the authors could more clearly conclude that POC birth testing for all children using specifically purchased devices may not be cost-effective; however, in x, y, z settings this approach is cost-effective. It would provide much clearer conclusions and suggestions for future guidance.

We were cautious with the interpretation in the original submission given that our primary effectiveness outcome does not have a defined willingness to pay threshold. We hope the added life-years gained threshold and sensitivity analyses have made the practical implications of our study stronger. We also added following to the discussion and point to our concluding paragraph which recommends countries with low transmission rates consider targeted VEID based on risk criteria (this is something we are currently evaluating in a related ongoing study).

> *Discussion*: Our analysis indicates that the survival benefits required for VEID to be considered cost-effective, 16–28 life-years per early ART initiation using cost-effectiveness thresholds from Pichon-Riviere et al. [21] or 6–8 life-years using GDP-based thresholds, are substantially higher than those demonstrated to date. In the CHER trial, which remains the primary benchmark [14], early versus deferred ART was associated with roughly 0.8 life-years gained over five years, based on reported deaths and follow-up time per arm. However, CHER compared 6-week to median 7-month ART initiation, while our study compared birth to 4–8-week ART initiation. Importantly, HIV-related infant mortality peaks around 2–3 months [5], suggesting that ART at birth could be underestimated. While early mortality reductions in the LIFE study were unfortunately not sustained through 18 months, most infants received LPV/r and viral suppression was low and associated with poor clinical outcomes overall [15]. With dolutegravir-containing ART now available for infants and expected to address common barriers to adherence due to once daily dosing and improved palatability, greater longer-term health benefits may be achievable than observed in earlier cohorts. Differences in comparator, follow-up, and treatment context caution against directly extrapolating CHER outcomes to our setting and highlight that long-term benefits of VEID are still uncertain.

...
Conclusion: Our analysis suggests that cost-effectiveness depends on whether ART initiation at birth translates to substantial long-term health benefit or if VEID can be targeted to infants at high risk of HIV acquisition. In settings with high vertical transmission rates or where weak HIV programs lead to significant gaps in infant retention in care, EID programs could consider adding PoC birth testing with careful consideration of PoC infrastructure utilization to improve affordability. In settings with low vertical transmission rates, the marginal benefits of universal birth testing are limited and targeted testing of high-risk infants at birth may be a more practical approach.

Comment 9

The last sentence of the second paragraph in the Abstract is duplicative. Instead some methodology on cost-effectiveness could be included.

We changed this sentence to:

Cost-effectiveness of VEID was evaluated relative to SoC with respect to ART initiation in the first week of life using Bayesian hierarchical models to calculate incremental cost-effectiveness ratios (ICERs).

Comment 10

Lines 222-223: retention in care, adherence to treatment, follow-up HIV testing are not linked or caused by POC EID. This sentence may need revision. Authors should be careful not to suggest this link throughout. Yes, POC can help with linkage to treatment, but thereafter it's about the system and services.

We meant to convey that to improve longer-term health outcomes, early ART interventions do not stop at birth. This sentence now reads:

Retention in care, adherence to treatment, and ensuring follow-up HIV testing for infants while they remain at risk for acquiring HIV, which depend on strong health systems and support services, are other important considerations when evaluating the cost-effectiveness of PoC EID approaches.

Comment 11

Lines 229-231: highlight that all infants testing HIV-negative should receive follow-up testing, not just those with a birth test.

Thank you for pointing out this error. We removed this.

Original submission
Additionally, all infants testing HIV-negative at birth should receive follow-up testing to identify those acquiring HIV during the breastfeeding period [3,16]...

Changes to the manuscript
Additionally, all infants should receive follow-up testing to identify those with undetectable virus at birth or acquiring HIV during the breastfeeding period [3, 16]...

Response to Reviewer 3

This paper presents important cost data on early infant diagnosis. The authors present the challenges well. Clearly earlier identification and treatment of HIV is a public health goal but also a moral obligation. That said, the authors identify the costs associated with early identification. The authors raise the important point that early ART initiation should lead to reduced mortality and the many other steps after diagnosis at birth that need to occur for this to happen- not easy but an important goal. One also wonders about the potential for cure if infants can be started early and appropriately virally suppressed. (https://www-nature-com.libproxy.lib.unc.edu/articles/s41591-024-03105-4)

Thank you for the positive evaluation. A leading argument for birth testing (and immediate treatment) is the potential for (functional) cure as suggested by the Bengu Nature Medicine paper and others.

Comment 1

Line 135- do reasons for not starting ART vary by arm..same comments for line 137 do reasons for delayed treatment vary by arm- imagine it would?

In the VEID arm (birth + 4-8 week tests), one infant did not start ART due to death on day 1 shortly after diagnosis, 1 had a delay in confirmatory testing which delayed ART initiation, and two delayed ART due to insufficient weight per guidelines. In the SoC arm (4-8 week test only), six infants were diagnosed retrospectively using stored DBS from birth and did not start ART due to death or loss to follow-up after birth. There were no delays in ART initiation in the SoC arm. Reasons for delayed or lack of treatment initiation were not key to this cost-effectiveness analysis and were already published previously [8], so we did not include further detail.

Comment 2

Line 144- "cost per infant was less than cost per test for SOC due to loss to follow up"- while this is real- it doesn't capture the real world "cost" of a child who is exposed and may have HIV and lost to follow up- it seems sensitivity analyses to address 'survivorship' bias here would be helpful? Inverse probability of treatment weights or imputation? It feels like part of the point of testing at birth is also to capture people while they are at the facility after birth- clearly capturing infections earlier but also capturing people who may not come back which clearly the SOC arm experienced.

In response to your concern about survivorship bias, we note that our main cost analysis reflects observed programmatic costs: VEID infants received testing at birth plus 4-8 weeks if they returned, while SoC infants only incurred costs if they returned for the 4-8 week test. Infants lost to follow-up in the SoC (about 5% of infants) therefore generated zero cost, which is operationally accurate but results in lower per-infant cost in the SoC. HIV-status was retrospectively ascertained for all infants, including those lost to retention. The difference in *1-week ART initiation* between arms is therefore structural rather than a survivorship artifact: by study design, no infants in the SoC had the opportunity to initiate ART in the first week, and the few who might have returned could not have met that outcome anyway. The *8-week testing outcome* is only minimally affected by loss to retention since loss to follow-up at 4-8 weeks was only about 5%.

Response to the Editor

We carefully checked the general editorial comments and confirm that:
1. We have reminded co-authors to submit a contributing author statement.
2. All figures have been checked with PACE and re-uploaded as high resolution TIFF files.
3. Data sharing statement is accurate; we confirm that all data used in the manuscript will be available either in the manuscript/supplement or upon request to a central data manager for sensitive health data.
4. All code will be available under: 10.5281/zenodo.14959301. We've added this link to our data sharing statement.
5. Financial disclosures, COI, DAS, and ethics statements are correct.
6. The study is reported according to CHEERS and the checklist has been uploaded as Supporting Information with section and page numbers.
7. Abstract adheres to PLOS Medicine headings.
8. Title is per PLOS Medicine style.
9. Non-technical Author Summary has been included.
10. Main results are expressed with 95% CIs and p-values where appropriate.
11. Page and line number are included in the manuscript file.
12. Citations are now in square brackets preceding punctuation.
13. All figures and tables have legends.
14. All abbreviations used in figures and tables have been defined in figure legends.
15. Red and green have been largely avoided and are never present in the same figure.
16. All comments pertaining to the Supplementary Material have been addressed.
17. Supporting information has been cited as suggested.
18. References are cited according to PLOS style.

References

[1] D. t. Brink, R. Martin-Hughes, A. L. Bowring, *et al.*, "Impact of an international hiv funding crisis on hiv infections and mortality in low-income and middle-income countries: A modelling study," *Lancet HIV*, 2025.

[2] UNAIDS, *Unaids data 2023*, 2024.

[3] C. De Schacht, H. Hoffman, N. Mabunda, C. Lucas, C. Alons, and A. Madonela, "High rates of hiv seroconversion in pregnant women and low reported levels of hiv testing among male partners in southern mozambique: Results from a mixed methods study," *PLoS One*, vol. 9, no. 12, e115014, 2014.

[4] J. Manyahi, B. Jullu, M. Abuya, J. Juma, J. Ndayongeje, and B. Kilama, "Prevalence of hiv and syphilis infections among pregnant women attending antenatal clinics in tanzania," *BMC Public Health*, vol. 15, p. 501, 2015.

[5] I. Sabi, H. Mahiga, J. Mgaya, O. Geisenberger, S. Kastner, and W. Olomi, "Accuracy and operational characteristics of xpert human immunodeficiency virus point-of-care testing at birth and until week 6 in human immunodeficiency virus-exposed neonates in tanzania," *Clinical Infectious Diseases*, 2019. DOI: 10.1093/cid/ciy538.

[6] M. F. Cotton, A. Violari, K. Otwombe, *et al.*, "Early time-limited antiretroviral therapy versus deferred therapy in south african infants infected with hiv: Results from the children with hiv early antiretroviral (cher) randomised trial," *Lancet*, vol. 382, no. 9904, pp. 1555–1563, Nov. 2013. DOI: 10.1016/S0140-6736(13)61409-9.

[7] A. Pichon-Riviere, M. Drummond, A. Palacios, S. García-Martí, and F. Augustovski, "Determining the efficiency path to universal health coverage: Cost-effectiveness thresholds for 174 countries based on growth in life expectancy and health expenditures," *The Lancet Global Health*, vol. 11, no. 6, e833–e842, 2023. DOI: 10.1016/S2214-109X(23)00162-6.

[8] I. Jani, I. Sabi, K. Elsbernd, *et al.*, "Impact of point-of-care birth test-and-treat on clinical outcomes among infants with hiv: A cluster-randomized trial in mozambique and tanzania," *Clinical Infectious Diseases*, vol. 80, no. 5, pp. 1114–1124, 2025. DOI: 10.1093/cid/ciae530.
