# Supplementary material for: Point-of-care early infant HIV diagnosis at birth in a pragmatic cluster-randomized trial in Mozambique and Tanzania: A comparative cost and cost-effectiveness study
Source: PLoS Med. 2026 May 6;23(5):e1005069. doi: 10.1371/journal.pmed.1005069 (PMC13166934; doi:10.1371/journal.pmed.1005069)
Supplement: Attachment — Submitted filename: Review_response_PlosMed__R2.pdf [file pmed.1005069.s006.pdf]

Author's responses to editors' and reviewers' comments for manuscript PMEDICINE-D-25-00626R2

Comparative cost and cost-effectiveness of point-of-care early infant HIV diagnosis at birth: Findings from a pragmatic cluster-randomized trial in Mozambique and Tanzania

Kira Elsbernd, Issa Sabi, Ilesh Jani, Chishamiso Mudenyanga, Siriel Boniface, Arlete Mahumane, et al.

Note: Editors' and Reviewers' comments are typeset in grey boxes and changes to the manuscript and supplement are typeset in yellow boxes. Rephrased or added text is typeset in blue. The respective changes in the tracked changes version of the manuscript are highlighted to indicate changes.

Note: During preparation of the final code release, we noticed a reproducibility issue in some results. To ensure reproducibility, minor changes have been made to some results. The overall conclusions of the manuscript remain the same.

Response to the Academic Editor

> The aim of the paper could be made more clear–i.e. to look at birth plus 4-6 week testing versus 4-6 week testing only, and that thus the used time horizon is appropriate as any assumptions after 6 weeks would be the same in both arms. There is interest in knowing what the addition of a birth HIV tests adds to getting HIV infected infants on ART as soon as feasible and thus reduce mortality - all the more important now, with reduced funding and consequent impact on health care/systems, when pregnant women may have reduced HIV testing and ART uptake and thus more likely to have vertically infected children.

Thank you for the comment. We have clarified the aim in the introduction and added a sentence justifying the time horizon to the methods as shown below.

> We estimated the cost of offering PoC EID and immediate ART under routine conditions to HIV-exposed infants at birth plus 4–8 weeks of age (very early infant diagnosis; VEID) compared with the standard-of-care (SoC) at 4–8 weeks of age only. We further assessed cost-effectiveness of VEID compared with the SoC with respect to early ART initiation and EID uptake, which are epidemiologically important intermediate outcomes on the causal pathway to reduced early infant mortality [14].
>
> ...
>
> The time horizon for individual-level cost and effectiveness outcomes corresponded to the 12-week follow-up period, during which clinical pathways and resource use differed between arms. Beyond this period, costs and clinical management were assumed to be equivalent in both arms.

Response to Reviewer 4

Comment 1

The study's outcomes (ART initiation and EID uptake) are intermediate process indicators rather than final health outcomes. The authors should clarify that this represents a cost-consequence or cost-effectiveness analysis using intermediate outcomes, not a full cost-utility or cost-effectiveness analysis based on health outcomes (e.g., DALYs or QALYs).

We added explicit mention of the study using an intermediate process outcome which is on the causal pathway to reduced mortality [1] to the final sentence of the introduction as well as the limitations, and point to the note on cost-utility analysis being beyond the scope of this manuscript in the limitations.

> We further assessed cost-effectiveness of VEID compared to the SoC with respect to early ART initiation and EID uptake, which are intermediate outcomes on the causal pathway to reduced infant mortality [14].
>
> ...
>
> Given the absence of a significant difference in longer-term health outcomes in the LIFE study [15], we restricted our analysis to a 12-week time horizon and assessed cost-effectiveness using intermediate outcomes relevant to EID programs as key indicators of health benefit.
>
> ...
>
> This approach excludes explicit examination of cost-utility in terms of quality- or disability-adjusted life years.

Comment 2

The study applies empirically derived country-level thresholds and GDP per capita values as comparators. However, these thresholds are expressed as cost per life-year gained, not per ART initiation. This mismatch should be explicitly acknowledged, as it limits direct interpretability of the ICERs presented.

We added the appropriate denominators for WTP thresholds to the Methods. We also added a clause to the Discussion clarifying the use of WTP thresholds to contextualize results and acknowledging the limitations of our methodology in directly interpreting the ICERs presented.

> We compared ICERs to empirically derived country-level cost-effectiveness thresholds expressed as cost per life-year from Pichon-Riviere et al. [22], which are based on health expenditure and life expectancy growth. These were $189 per life-year in Mozambique and $316 per life-year in Tanzania (2020 US$). For comparability with previous studies, we also considered GDP per capita as a willingness to pay (WTP) benchmark ($462 in Mozambique and $1117 in Tanzania per life-year gained).
>
> ...
>
> We compared ICERs to empirically derived cost-effectiveness thresholds as well as GDP per capita [21, 22, 38], however, these WTP thresholds, expressed in terms of life-years gained rather than additional early ART initiations, should be used to contextualize results and not to directly interpret ICERs.

Comment 3

While "ART initiation within one week" is programmatically important, its link to health benefit is indirect. Without estimating life-years gained, ICERs per early ART initiation are difficult to interpret. The threshold analysis is creative but cannot substitute for a formal utility-based analysis.

We agree that life-years or DALYs are the preferred health outcomes for cost-effectiveness or cost-utility analysis when robust evidence on long-term effects are available. However, in this trial-based evaluation, modeling life-years or DALYs would require strong assumptions that are not supported by the current evidence base.

Specifically, the LIFE study was not powered to detect differences in mortality alone (the primary endpoint was a composite endpoint covering mortality, morbidity, treatment failure, and loss to follow-up broadly), and no significant differences in mortality (or the composite endpoint) were observed during the follow-up period. Beyond the LIFE study, the strongest evidence comes from the CHER trial [1], which compared ART initiation at 6 weeks with a median of 7 months of age. These data also do not directly inform the incremental survival benefit of starting ART at birth compared to 4-8 weeks of age, which is the comparison evaluated in this study.

In our context, extrapolating life-years gained or DALYs averted would require strong assumptions about mortality hazards, treatment effects, and long-term retention that cannot be empirically validated at this point. We focused on early ART initiation as an epidemiologically meaningful intermediate outcome which is on the causal pathway to reduced early infant mortality.

We added the threshold analysis estimating the minimum life-years gained per additional infant initiating ART in the first week of life that would be needed to reach cost-effectiveness thresholds to support the interpretation of ICERs. We also contextualized these results using survival estimates from CHER in the Discussion, while explicitly acknowledging the limitations of extrapolating these findings to our birth to 4-8-week comparison.

We have clarified this rationale in the Methods and Discussion.

Original submission

To link our 1-week ART initiation outcome to longer-term health gains, we estimated the life-years gained that would be needed for our intervention to be considered cost-effective.

Changes to the manuscript

In the absence of robust data quantifying the incremental survival benefit associated with starting ART in the first week of life relative to starting at 4-8 weeks of life, we estimated the life-years that would need to be gained per additional ART initiation within one week for our intervention to be considered cost-effective given standard cost-effectiveness thresholds.

...

Rather than model long-term health outcomes directly, we derived the number of life-years that would need to be gained per additional infant initiating ART within one week of life for VEID to be considered cost-effective, using posterior draws of incremental cost and incremetnal effect from the 1-week ART model to propagate parameter uncertainty. Results are reported as median and 95% CrI.

...

The life-years estimates above are a derived benchmark, intended to contextualize the magnitude of survival benefit required for cost-effectiveness, and are assessed against findings from the CHER trial.

...
Our life-years estimate provided an informative benchmark of the survival benefits required for cost-effectiveness, but these estimates were model-based and depend on assumptions about WTP thresholds.

Comment 4

The manuscript reports ICERs (e.g., $3,000 per additional early ART initiation) and subsequently infers that 16-28 life-years gained per early initiation would be required for cost-effectiveness. This calculation reflects a reverse application of cost-effectiveness thresholds rather than a modelled ICER per life-year gained. The authors should clearly state that this is a threshold-based calculation, not a modeled estimate, and present the formula linking incremental cost per ART initiation to the implied cost per life-year gained.

We included the threshold analysis to support the interpretation of ICERs, and have labeled this as a derived benchmark throughout. We also added the formula to the Supplement (Section 5.7) as suggested.

Comment 5

The 12-week analytic horizon is too short to capture meaningful health and cost outcomes of early ART. Although the authors estimate the required life-years for cost-effectiveness, this remains a post hoc conceptual exercise rather than a formal model. The limitations of this short horizon should be discussed explicitly, and the potential value of a decision-analytic or Markov extrapolation acknowledged.

We agree that the 12-week time horizon does not capture long-term health outcomes of early ART. The analytic period was intentionally aligned with the period over which the intervention (addition of test and treat at birth) alters clinical pathways. Beyond this period, clinical care processes and costs were assumed to be the same across arms. We added the following to the Methods and Discussion, respectively.

The time horizon for individual-level cost and outcomes corresponded to the 12-week follow-up period, during which clinical pathways and resource use differed between arms. Beyond this period, costs and clinical management were assumed to be equivalent in both arms.
...
Given the absence of a significant difference in longer-term health outcomes in the LIFE study [15], we restricted our analysis to a 12-week time horizon and assessed cost-effectiveness using intermediate outcomes relevant to EID programs as key indicators of health benefit.

Comment 6

The study adopts a healthcare provider perspective, excluding patient and societal costs. The statement that patient costs were "minimal and non-monetary" lacks empirical justification. Please provide evidence or references supporting this assumption, or discuss its limitations.

Thank you for pointing this out. We have softened the language and point to the limitation that direct patient costs are beyond the scope of this work.

> Given that we did not systematically document direct client costs in our study, we focused on health system costs which are practical to capture and reflect the expenditures most relevant for planning and resource allocation.
> ...
> Finally, direct patient and broader societal costs (e.g., schooling gains) were beyond the scope of this work.

Comment 7

The exclusion of confirmatory central laboratory testing and potential shared resource allocation may underestimate total costs. The method for apportioning shared costs (e.g., electricity, space, analyzer use for other assays) should be described more clearly.

Confirmatory testing at the central laboratory represented only 1.3% of all EID tests in the study and as we did not have price data, we excluded it as it would have a negligible impact on the results. We put this information in the supplement. We also added the following to the Methods to clarify the costing of other shared resources.

> *Changes to the supplement*
> Cost estimates for central laboratory-based testing for resolution of discrepant results or in case reagents were not available at the sites were not available and therefore were not included. However, central laboratory-based testing represented only 1.3% of all EID tests in the LIFE study and costs are typically less than PoC EID testing costs [6].
>
> *Changes to the manuscript*
> Shared resources (e.g., analyzers, utilities, and communication) were allocated to VEID proportionally based on observed PoC EID testing volume relative to total analyzer use.

Comment 8

Details of the Bayesian hierarchical (hurdle Gamma) model, priors, and convergence diagnostics are only provided in the supplement. A concise summary of the model structure, priors, and diagnostics ($\hat{R}$ values, trace plots, posterior predictive checks) should be included in the main text for transparency.

We added a summary of model structure, priors, and convergence diagnostics to the Methods and retained full details in the Supplement.

> Incremental costs and incremental effectiveness were estimated using multivariate Bayesian hierarchical models to account for skewed cost data and clustering at the health facility level, with results reported as posterior mean and 95% credible interval (CrI). Costs were modeled on the natural scale using a hurdle Gamma specification to accommodate structural zeros arising from

infants who did not receive EID testing due to loss to follow-up, while effectiveness outcomes were modeled as Bernoulli processes. Models were fit separately for 1-week ART and 8-week PoC EID effectiveness outcomes and included country- and site-level random effects to capture intra-cluster correlation and differences in costs, PoC testing platforms, prophylaxis protocols, and transmission rates. Weakly informative priors were specified throughout to reflect expected differences between VEID and SoC arms. Model convergence was confirmed (all $\hat{R} \approx 1$), and posterior predictive checks indicated good agreement between observed and replicated data. Full model specification, priors, and diagnostics are provided in S1 Supplementary Methods.

Comment 9

The reported posterior correlation ($\rho = -0.25$) is not interpreted. The authors should clarify what this correlation indicates about the relationship between costs and effects.

Thanks for pointing out this oversight. ρ in this instance is a correlation between cost random effects, specifically the posterior mean correlation between site-level baseline costs and the marginal cost increase associated with increased testing volume. We added the following sentence to the Results.

Sites with higher baseline costs tended to experience smaller increases in cost per additional test as testing volumes increased (posterior correlation $\rho = -0.25$), consistent with economies of scale.

Comment 10

The classification of zero-cost cases (infants lost to follow-up) as structural zeros should be explicitly justified, as this may bias mean cost estimates downward.

Thank you for this comment. While incorporating structural zeros may mathematically bias cost per infant downward compared to a scenario of perfect retention (i.e., efficacy-type analysis), our analysis is designed as a pragmatic, effectiveness-based evaluation intended to inform real-world implementation. The loss to follow-up is a reality of service delivery in this context. We chose not to include a sensitivity analysis with modeled costs and outcomes for infants lost to follow-up as the probability of zero cost cases was only 5% and it requires assumptions about the missingness mechanism and risks introducing structural uncertainty.

We added the following to the Methods and Discussion, respectively.

Infants lost to follow-up after birth in the SoC arm incurred no intervention-related resource use and were therefore treated as structural zeros with respect to costs.

...

Additionally, all infants should receive follow-up testing to identify those with undetectable virus at birth or acquiring HIV during the breastfeeding period [3, 16], especially considering potential disruptions to maternal ART access due to funding cuts. We assumed that infants lost to follow-up did not incur further program-related costs. If unobserved services were received elsewhere, this could bias costs downward. However, loss to follow-up was low (<5%) in our study. While full

population coverage and universal follow-up testing would slightly increase program costs, our estimates reflect what a well-run program could realistically achieve.

Comment 11

The manuscript does not specify whether intra-cluster correlation (ICC) was accounted for in bootstrapping or Bayesian estimation. ICCs for both cost and effect outcomes should be reported, and the handling of clustering in ICER estimation should be clarified.

We did account for intra-cluster correlation in the modeling and have clarified the inclusion of random effects in the Methods. Due to model instability, we were unable to include site-level random intercepts in the 1-week ART outcome model and instead adjusted for country through a fixed effect. We've added ICCs computed from posterior draws for cost and PoC EID uptake outcomes to the Results.

Site-level variation was modest with an intra-cluster correlation of 0.033 (95% CrI: 0.018–0.059) for cost per infant and 0.140 (0.066–0.258) for PoC EID within eight weeks.

Comment 12

The use of empirically derived thresholds is appropriate, but the link between these thresholds and local opportunity costs in Mozambique and Tanzania should be better justified. A short discussion on their applicability for decision-makers would strengthen the policy relevance.

Thank you for this comment. We added the following to the Discussion.

The cost-effectiveness thresholds used are intended to approximate health system opportunity costs and should be considered alongside local budget constraints, feasibility, and equity priorities when informing policy decisions.

Comment 13

The probabilistic sensitivity analysis (PSA) lacks detail regarding which parameters were varied (e.g., costs, clinical inputs). Please clarify.

We clarified in the Methods that the PSA reflects posterior uncertainty from the Bayesian models.

We assessed uncertainty in ICERs using probabilistic sensitivity analyses reflecting posterior uncertainty in costs and effectiveness outcomes derived from the Bayesian hierarchical models.

Comment 14

The manuscript states that costs were converted to 2020 USD; please detail the inflation adjustment process and indices used (2020-2021).

We used annual mean exchange rates from the World Bank GEM database to convert local currency to nominal 2020 US$. We did not adjust for inflation across years since it would have had negligible impact on the results given the single-year focus (majority of costs collected in 2020) and short time-frame. This is in line with World Bank cost studies which commonly report nominal US$ for the year of spending. We added explicit mention of nominal US$ to the Methods and added detail to the Supplement to clarify the choice.

Comment 15

Include exchange rate and inflation details in the main text rather than only in the supplement.

We added the rates used to the main text.

Comment 16

Clearly define the numerator and denominator of the ICERs (e.g., per HIV-exposed or HIV-positive infant).

We have clarified the denominators as follows in the Methods.

> Incremental costs, incremental effectiveness, and cost-effectiveness ratios (ICERs) per additional infant initiated on ART within one week and per additional infant receiving a PoC EID test within eight weeks were estimated relative to the SoC.

Comment 17

Ensure consistency between the text and CHEERS checklist—some items (e.g., 12 and 17) lack sufficient detail in the manuscript.

The clarifications and changes made based on the other comments now satisfy the CHEERS items. We added detail to the measurement of outcomes (item 12) and model validation details to the statistical analysis section (item 17).

Comment 18

Given the discussion around high-risk infants, consider addressing equity or distributional aspects of cost-effectiveness.

Thank you for this comment. We added a brief discussion of equity considerations, focusing on differential benefits for high risk infants and health facility capacity.

Where and to whom VEID is offered may also have important equity implications. The benefits of VEID are likely concentrated among infants at higher risk of vertical transmission and in health facilities with sufficient capacity to reliably deliver timely testing and treatment. In settings with lower testing volumes or weaker follow-up systems, costs and benefits may differ, underscoring the importance of pairing VEID implementation with health system strengthening so that it reduces disparities in infant HIV testing and treatment.

Comment 19

Maintain consistent terminology (e.g., use "95% CrI" for Bayesian intervals and "incremental cost per additional infant initiating ART within 1 week" throughout).

We reviewed and cleaned up the terminology throughout.

Comment 20

The joint Bayesian model combines cost and effect outcomes, but diagnostics (e.g., posterior predictive checks, residual plots) are only briefly mentioned in the supplement. Include a short summary of model validation.

We added this to the Methods (see also the response to Comment 8).

Comment 21

The deterministic sensitivity analysis ranges (Figure 6) appear arbitrary—indicate whether these are empirical, elicited, or assumed (e.g., ±25%).

We added justification for the ranges used to the Methods.

Deterministic sensitivity analyses were utilized to explore the impact of selected drivers of cost per test not explicitly parameterized in the Bayesian hierarchical models. We chose ranges reflective of plausible uncertainty and policy-relevant scenarios. Consumable prices were varied by ±50% to account for uncertainty in procurement and distribution costs, testing volume from -50% to 200% of observed levels to reflect variability across sites and potential scale-up, analyzer lifespan from 4 to 15 years for a conservative lower bound and assumptions that PoC platforms can last longer with maintenance, and discount rate from 0 to 6% in line with standard guidance [24]. ...

Comment 22

The probabilistic framework does not appear to propagate uncertainty from all cost inputs. Broader PSA incorporating input uncertainty (e.g., consumables, lifespan, capital costs) would be expected in a full CEA.

As this was a trial-based economic evaluation, uncertainty was propagated through posterior draws from the Bayesian models, which captures joint uncertainty and correlation between costs and effectiveness outcomes at the individual level as well as site level. We clarified this in the Methods and added a sentence to the limitations.

> We assessed uncertainty in ICERs using probabilistic sensitivity analyses reflecting posterior uncertainty in costs and effectiveness outcomes derived from the Bayesian hierarchical models.
> ...
> Our probabilistic sensitivity analysis propagates uncertainty arising from observed variation in costs and effectiveness outcomes captured by the Bayesian hierarchical models. We did not additionally model uncertainty in individual cost inputs, as the analysis was based on observed resource use and expenditures.

Comment 23

The CEACs (Figure 4) should explicitly label axes and clarify their relationship to willingness-to-pay thresholds.

We re-labeled CEAC axes as "Willingness to pay (2020 US$; per life-year)" and "Probability VEID cost-effectives versus SoC" in Figure 4.

Comment 24

The use of p-values (e.g., p=0.112, p<0.0001) is inconsistent with a Bayesian analysis. Either justify the hybrid frequentist approach or replace these with Bayesian credible intervals.

We added a sentence clarifying that frequentist methods were used for descriptive and design-based comparisons. We also removed largely unnecessary p-values from the first section of the Results to focus on effect sizes and timing and improve consistency with the descriptive purpose of these analyses and the Bayesian framework used for ICER estimation.

> Frequentist methods were used for descriptive summaries and unadjusted comparisons between study arms.

Comment 25

For clarity, replace "ICER per 1-week ART initiation" with "incremental cost per additional infant initiating ART within 1 week."

We aligned terminology in Figure 7 and throughout.

Response to the Editor

* Please confirm that your title complies with PLOS Medicine's style. Your title must be nondeclarative and not a question. It should begin with main concept if possible. "Effect of" should be used only if causality can be inferred, i.e., for an RCT. Please place the study design ("A randomized controlled trial," "A retrospective study," "A modelling study," etc.) in the subtitle (ie, after a colon).
* In the last sentence of the Abstract Methods and Findings section, please describe the main limitation(s) of the study's methodology.
* In the last bullet point of the Author study, please add the study limitations in very general language.
* In figures 3 and 6, please change one of the blue colours in the figures for improved contrast.

We confirm that the title complies with PLOS Medicine's style.
We added limitations to the Abstract and Author Summary.
The blue colors in the Figures 3 and 6 have been adjusted to improve contrast.

References

[1] M. F. Cotton et al., "Early time-limited antiretroviral therapy versus deferred therapy in south african infants infected with hiv: Results from the children with hiv early antiretroviral (cher) randomised trial," *Lancet*, vol. 382, no. 9904, pp. 1555–1563, Nov. 2013. DOI: 10.1016/S0140-6736(13)61409-9.
