# Supplementary material for: Point-of-care early infant HIV diagnosis at birth in a pragmatic cluster-randomized trial in Mozambique and Tanzania: A comparative cost and cost-effectiveness study
Source: PLoS Med. 2026 May 6;23(5):e1005069. doi: 10.1371/journal.pmed.1005069 (PMC13166934; doi:10.1371/journal.pmed.1005069)
Supplement: Attachment — Submitted filename: Review_response_PlosMed__R3_.pdf [file pmed.1005069.s007.pdf]

Author's responses to editors' and reviewers' comments for manuscript PMEDICINE-D-25-00626R3

Comparative cost and cost-effectiveness of point-of-care early infant HIV diagnosis at birth: Findings from a pragmatic cluster-randomized trial in Mozambique and Tanzania

Kira Elsbernd, Issa Sabi, Ilesh Jani, Chishamiso Mudenyanga, Siriel Boniface, Arlete Mahumane, et al.

Note: Editors' and Reviewers' comments are typeset in grey boxes and changes to the manuscript and supplement are typeset in yellow boxes. Rephrased or added text is typeset in blue. The respective changes in the tracked changes version of the manuscript are highlighted to indicate changes.

Response to the Editor

> Please confirm that your title complies with PLOS Medicine's style. Your title must be non-declarative and not a question. It should begin with main concept if possible. "Effect of" should be used only if causality can be inferred, i.e., for an RCT. Please place the study design ("A randomized controlled trial," "A retrospective study," "A modelling study," etc.) in the subtitle (ie, after a colon). We suggest: "Point-of-care early infant HIV diagnosis at birth in a pragmatic cluster-randomized trial in Mozambique and Tanzania: a comparative cost and cost-effectiveness study"

We changed the title to the suggested one.

> Abstract:
> - Please confirm that your abstract complies with our requirements, including format (three sections: Background, Methods and Findings, and Conclusions) and providing all the information relevant to this study type
> - In the last sentence of the Abstract Methods and Findings section, please describe the main limitation(s) of the study's methodology. Please clarify in the Abstract whether this was a prespecified secondary analysis.

- We confirm that the abstract complies with the requirements.
- We revised the final sentence of the Methods and Findings section to explicitly state the limitation.
- We added that this was a pre-specified secondary objective of the LIFE study.

> This pre-specified secondary analysis was...
>
> ... These findings are limited by the lack of long-term health outcome data and reliance on an intermediate outcome; therefore, we estimated that VEID would need to yield 6-32 life-years gained per additional early ART initiation to be considered cost-effective.

Author Summary:
- In the author summary, in the final bullet point of 'What Do These Findings Mean?', please include the main limitations of the study in non-technical language.
- The second section of the Author summary should be titled "What did the researchers do and find?"
- The text of the Author Summary is subject to editorial change and should be distinct from the scientific abstract.

We revised the final bullet point of the author summary for clarity and corrected the section title.

A limitation of our study is that infants were followed only for the first few months of life, meaning we could not directly measure long-term health benefits of starting treatment earlier. Instead, we estimated the minimum survival benefit required (6–32 additional years of life) for be for VEID to be considered cost-effective at current prices.

Main text:
- Please ensure that the Introduction ends with a clear description of the study question or hypothesis.
- Please ensure that all abbreviations are defined at first use throughout the text.
- Please confirm that all numbers presented in the abstract are present and identical to numbers presented in the main manuscript text.
- Please revise for use of patient-centered language. Please note that patient-centered language is constructed with the use of post-modified nouns (e.g. 'patients with psoriasis' (or similar) instead of 'psoriasis patients') putting the person first in the sentence structure.

- We revised the last 2 sentences of the introduction to explicitly state the study objective.

The objective of the study was to estimate the cost and cost-effectiveness of offering PoC EID and immediate ART to infants exposed to HIV under routine conditions at birth plus 4–8 weeks of age (very early infant diagnosis; VEID) compared with the standard-of-care (SoC) at 4–8 weeks of age only. Cost-effectiveness was evaluated with respect to...

- We confirm that all abbreviations are defined at first use.
- We confirm that all numbers are present and identical to numbers in the main text.
- We have reviewed and revised for people-first language throughout.

Statistical reporting: Please revise throughout the manuscript, including tables and figures.
- Please report statistical information as follows to improve clarity for the reader ""22% (95% CI [13,28]; p</=)"". CI ranges in your manuscript need to expressed with commas, not hyphens.
- Please separate upper and lower bounds with commas instead of hyphens as the latter can be confused with reporting of negative values.
- Please repeat statistical definitions (HR, CI etc.) for each set of parentheses.

We revised the statistical reporting to match the requested style throughout.

Figures:
- Please define all elements of box plots in the figure caption - center line, box limits and whiskers.
- Please convert any stacked bar charts to another data representation for example a table, or other type of graph. Figures 3 and S2 contain stacked bar graphs. If these cannot be converted to another format, we require that you add the underlying data to a table for each figure in the supplementary material.

- We defined the box plot elements in the Figure 2 caption.
- We added a table to the supplement (Table S1) showing the underlying data for Fig 3 and Fig S2.

Discussion:
- Please remove the 'conclusions' subheading from the discussion.

"Conclusion" has been removed.

Acknowledgments:
- Please include an Acknowledgments section in your manuscript that recognizes study participants and individuals who played a role in data collection or participant care or involvement.

Thank you for pointing out this oversight. We have added our acknowledgment section to the resubmission.

Disclosures:
- Please revise the COI statement in view of the fact that one of the authors (TB) is editor-in-chief of PLOS Medicine. For example, "TB is editor-in-chief of PLOS Medicine. The other authors declare no competing interests.
- The funding statement should include: specific grant numbers, initials of authors who received each award, URLs to sponsors' websites. Also, please state whether any sponsors or funders (other than the named authors) played any role in study design, data collection and analysis, the decision to publish, or preparation of the manuscript.
- It appears that one or more study authors is affiliated with one or more of the agencies that funded the study. Thus, the statement "The funders had no role in study design, data collection and analysis, decision to publish, or preparation of the manuscript" does not apply. Please revise the Financial Disclosure accordingly, as in "[Author name] is [author's role] at [funding agency]. The funders had no other role in study design. . . .."
- All authors must declare their relevant competing interests per the PLOS policy. For authors with ties to industry, please indicate whether any of the interests has a financial stake in the results of the current study.

We have revised the COI and Financial disclosures statements as requested.

- Please report your economic analysis according to the appropriate study design and provide the relevant completed checklist. In the checklist please include sufficient text excerpted from the manuscript to explain how you accomplished all applicable items.
- Please use sections rather than page numbers or lines in the checklist.

We have replaced page and line numbers with sections in the checklist.

- PLOS defines the "minimal data set" to consist of the data set used to reach the conclusions drawn in the manuscript with related metadata and methods, and any additional data required to replicate the reported study findings in their entirety. Authors do not need to submit their entire data set, or the raw data collected during an investigation. Please submit the following data:

The values behind the means, standard deviations and other measures reported;
The values used to build graphs;
The points extracted from images for analysis.
- Please confirm that the data in the manuscript adheres to the minimal data set requirements.
- The Data Availability Statement (DAS) requires revision given that the IPD is not included in the manuscript files. For each data source used in your study:

a) If the data are freely or publicly available, note this and state the location of the data: within the paper, in Supporting Information files, or in a public repository (include the DOI or accession number).
b) If the data are owned by a third party but freely available upon request, please note this and state the owner of the data set and contact information for data requests (web or email address). Note that a study author cannot be the contact person for the data.
c) If the data are not freely available, please describe briefly the ethical, legal, or contractual restriction that prevents you from sharing it. Please also include an appropriate contact (web or email address) for inquiries (again, this cannot be a study author).

- For studies in which a novel model is central to the manuscript's findings, authors are responsible for providing the source code needed to replicate the study's findings in a repository (such as GitHub, SourceForge or Bitbucket) or a cloud computing service (such as Code Ocean). Protection of authors' intellectual property will not be cause for exception. Please explain in the manuscript's Data Availability Statement how readers can access the shared code.

As stated in our data availability statement, cost input data is provided in the manuscript (Table 1), and individual-level health outcome data contain sensitive and potentially identifying information (due to small numbers of infants with HIV). We have provided a non-author contact for inquiries per item c) above. In addition, limited datasets (e.g., with sensitive data redacted, posterior draws) and code are available via the Zenodo link provided.

Response to Reviewer 4

Comment 1

Although the informative priors on the study arm effects (Normal(-5,1) and Normal(5,1)) are justified conceptually, no formal prior sensitivity analysis is presented. Given the strength of these priors and the relatively small number of HIV-positive infants, I recommend conducting sensitivity analyses using weaker priors (e.g., Normal(0,2.5) or similar) to demonstrate that posterior inferences are robust and not materially driven by prior assumptions.

Thank you for this suggestion. Posterior estimates of incremental effectiveness were consistent with the primary analysis using the weaker priors suggested, which confirms that results were primarily informed by the observed data rather than prior specification. Results of this sensitivity analysis were added to the Supplement (Table SM 2).

Comment 2

It is stated that no random effects were included in the 1-week ART initiation model. Given the cluster-randomised design and observed clustering in other outcomes, please clarify the rationale for excluding site-level random effects in this model. If the decision was based on negligible ICC, convergence issues, or limited events, this should be explicitly stated and supported.

The 1-week ART model did not include site-level random effects because the number of infants with HIV was small relative to the number of sites, with resulting instability in cluster-level variance estimation. In preliminary models with random effects, the probability of 1-week ART was similar. For model stability, we therefore used a fixed effect for country (representing the higher level clustering). We clarified this in the methods.

> For the 1-week ART model, site-level random effects were not included due to sparse outcome variation within clusters, which precluded reliable estimation of additional variance components. Country was included as a fixed effect to account for the systematic differences in context.

Comment 3

The ICER is calculated as the ratio of mean incremental cost to mean incremental effect. Please clarify whether ICERs were also evaluated at the posterior draw level (i.e., (i.e., $\Delta C_i/\Delta E_i$) and whether the chosen approach preserves the joint uncertainty structure between cost and effectiveness. If not, please justify the methodological choice.

Thank you for highlighting this point. To generate point estimates of ICERs, we used a ratio of means, but to explore uncertainty around these estimates, posterior samples of incremental cost and effect pairs were drawn to propagate uncertainty. The latter were used for deriving life-years required for cost-effectiveness and cost-effectiveness planes and acceptability curves. This is consistent with standard economic analysis. We've clarified in the methods.

ICER point estimates were calculated as the ratio of mean incremental cost to mean incremental effect from the posterior distributions. To reflect uncertainty around this estimate, we used posterior draws of incremental cost and incremental effect jointly sampled from the Bayesian hierarchical models in probabilistic sensitivity analyses. ...

Comment 4

The choice of a hurdle Gamma model for costs is reasonable, but the manuscript does not report whether alternative specifications (e.g., log-normal, zero-inflated Gamma) were considered. If model comparison was conducted (e.g., LOO-IC or WAIC), please report results; otherwise, please justify the final specification more explicitly.

While we considered alternative specifications for the cost model conceptually, we opted for a hurdle Gamma based on the data structure and standard econometric practice. First, we made the assumption that zeros in our data are structural (i.e., no resource consumption) rather than sampling zeros (i.e., costs not recorded). The hurdle model preserves the distinction between receiving EID testing (hurdle) and the "intensity" of the testing (Gamma) better than a zero-inflated model. Second, a log-normal can be overly sensitive to extreme cost outliers, of which our data contains few, leading to an overestimate of mean costs when back-transformed. We added a paragraph to the Supplement to justify the choice.

A hurdle Gamma specification was selected over alternative models (e.g., log-Normal or zero-inflated Gamma) because, from a provider perspective, the zeros in this study represent structural non-utilization outcomes rather than latent or missing sampling zeros; specifically, infants who did not receive EID testing at the study sites due to death or loss to follow-up between birth and study follow-up. Furthermore, a Gamma distribution with a log-link was chosen over a log-Normal specification because it avoids over-sensitivity to extreme outliers and back-transformation bias often found with Log-Normal OLS models. The appropriateness of this specification was confirmed via posterior predictive checks (Figure SM3).

Comment 5

The redistribution of HIV testing costs to HIV-positive infants relies on bootstrap-derived intrauterine transmission probabilities. While this approach is transparent, additional clarification on the epidemiological plausibility of the 0-40% sensitivity range would strengthen the robustness of this assumption.

In the study settings, UNAIDS estimates indicate that approximately 5% of HIV-exposed infants acquire HIV by 6 weeks, the closest population-level proxy for intrauterine transmission. We explored a wider range of early transmission probabilities to assess how cost-effectiveness of VEID would vary across epidemiological contexts with higher underlying transmission risk (for example, due to impacts of funding cuts to HIV programs). This analysis also allows identification of threshold transmission levels at which VEID would meet commonly used cost-effectiveness benchmarks.

We have clarified this rationale in the manuscript to emphasize that this sensitivity analysis serves as a threshold analysis of the transmission levels required for VEID to be considered cost-effective at

current costs and assuming that earlier ART initiation translates to long-term survival gains, rather than representing expected epidemiological conditions in the study settings.

> We additionally varied the intrauterine transmission rate across a wide range of values within a probabilistic sensitivity analysis framework to assess how cost-effectiveness would vary across epidemiological contexts and to identify threshold levels of transmission at which VEID would meet common cost-effectiveness benchmarks.
>
> ...indicating that VEID becomes cost-effective only above specific thresholds of early vertical transmission risk. These thresholds are based on cost per additional infant initiating ART within one week of life and do not directly incorporate downstream life-years gained, which are explored separately above.

Comment 6

The decision to benchmark required life-years gained against CHER trial results assumes transferability across settings and time periods. Please expand discussion of structural uncertainty related to differences in ART regimens, background mortality, adherence, and health system context.

Thank you for this suggestion. We added a the following in the discussion of the comparison to CHER.

> ... Differences in treatment regimens, adherence patterns, background mortality, and health system context across settings and time introduce structural uncertainty that may influence the magnitude of survival benefits achievable with ART initiation in the first week of life. ...

Comment 7

Please clarify explicitly the analytic perspective (e.g., health system perspective) and confirm that patient-incurred or societal costs were intentionally excluded.

We have clarified the analytic perspective and explicitly state that direct patient and societal costs were intentionally excluded.

> We conducted a micro-costing study from the health system perspective. We estimated implementation and operations costs as they occurred in the LIFE study, including fixed costs (equipment purchase, installation, and maintenance; utilities; communication; training; and facility upgrades) and variable costs (consumables, labor, and ART) for VEID and SoC approaches. Direct client and societal costs were excluded from this analysis, as the study was designed to focus on direct budgetary and resource allocation impact on the healthcare provider.

Comment 8

The rationale for limiting ART cost inclusion to 4 weeks (Mozambique) and 6 weeks (Tanzania) should be more clearly justified, including discussion of how this conservative assumption affects ICER interpretation.

ART costs were included only during the intervention period because treatment pathways beyond this period were assumed to converge. We added the following as justification to the Supplement.

By limiting the cost window to this period, we capture the specific incremental cost of "very early" ART initiation, i.e., the 4-6 weeks of ART that the intervention group receives treatment while the SoC group is still awaiting their first test. While this approach excludes long-term ART costs, it also excludes the potential high-cost clinical complications (e.g., hospitalizations) associated with delayed diagnosis in the SoC arm, which fall outside the scope of the study.

Comment 9

Capital cost lifespan assumptions (5-year platform, 10-year facility upgrades) materially influence annualized fixed costs. Consider briefly discussing how sensitive results are to these assumptions.

Sensitivity analysis varying analyzer lifespan from 4-15 years demonstrated moderate influence on cost per test (Figure 6 and S3). We did not vary the facility upgrades lifespan, however, these were not major cost drivers.

Comment 10

Since PoC platforms were shared with other services (particularly in Tanzania), please clarify how overhead and shared usage were allocated and whether alternative allocation rules were explored.

Shared resource allocation is mentioned in the methods, with further details provided in the Supplement. Alternative allocation rules were not explored in this manuscript.

Comment 11

It would be helpful to indicate whether prior-posterior overlap or prior predictive checks were examined for key parameters to demonstrate that inference is primarily data-driven.

We assessed the influence of prior assumptions as suggested and compared prior and posterior distributions for key parameters. This is now included in Table S1 and discussed in the supplement.

Comment 12

Consider briefly discussing structural uncertainty (e.g., alternative modelling frameworks or longer-term extrapolation approaches), even if not implemented.

Thank you for the suggestion. We added a sentence to the limitations.

... Dynamic, longer-term modeling frameworks could more explicitly account for longitudinal clinical transitions and age-dependent mortality, though such approaches would require substantial extrapolation beyond the observed data. ...
